# Factors influencing treatment decision‐making for cancer patients in low‐ and middle‐income countries: A scoping review

**DOI:** 10.1002/cam4.6375

**Published:** 2023-08-01

**Authors:** Marta Salek, Allison Silverstein, Alyssa Tilly, Pascale Yola Gassant, Sanjeeva Gunasekera, Diriba Fufa Hordofa, Donna Hesson, Caitlyn Duffy, Nauman Malik, Michael McNeil, Lisa M. Force, Nickhill Bhakta, Danielle Rodin, Erica C. Kaye

**Affiliations:** ^1^ Department of Global Pediatric Medicine St Jude Children's Research Hospital Memphis Tennessee USA; ^2^ Department of Pediatrics University of Colorado School of Medicine Aurora Colorado USA; ^3^ Division of General Medicine and Clinical Epidemiology University of North Carolina School of Medicine Chapel Hill North Carolina USA; ^4^ Department of Oncology Saint‐Damien Hospital Port‐au‐Prince Haiti; ^5^ Department of Paediatric Oncology National Cancer Institute Maharagama Sri Lanka; ^6^ Department of Pediatrics and Child Health Jimma University Medical Center Jimma Ethiopia; ^7^ Welch Medical Library Johns Hopkins University Baltimore Maryland USA; ^8^ Department of Radiation Oncology University of California, San Francisco San Francisco California USA; ^9^ Department of Health Metrics Sciences and Department of Pediatrics, Division of Pediatric Hematology/Oncology University of Washington Seattle Washington USA; ^10^ Department of Radiation, Oncology University of Toronto Toronto Ontario Canada; ^11^ Radiation Medicine Program Princess Margaret Cancer Centre Toronto Ontario Canada; ^12^ Canada Global Cancer Program Princess Margaret Cancer Centre Toronto Ontario Canada; ^13^ Department of Oncology St Jude Children's Research Hospital Memphis Tennessee USA

**Keywords:** decision‐making, low‐ and middle‐income countries, oncology, pediatric oncology, scoping review

## Abstract

**Purpose:**

In this scoping review, we evaluated existing literature related to factors influencing treatment decision‐making for patients diagnosed with cancer in low‐ and middle‐income countries, noting factors that influence decisions to pursue treatment with curative versus non‐curative intent. We identified an existing framework for adult cancer developed in a high‐income country (HIC) context and described similar and novel factors relevant to low‐and middle‐income country settings.

**Methods:**

We used scoping review methodology to identify and synthesize existing literature on factors influencing decision‐making for pediatric and adult cancer in these settings. Articles were identified through an advanced Boolean search across six databases, inclusive of all article types from inception through July 2022.

**Results:**

Seventy‐nine articles were identified from 22 countries across six regions, primarily reporting the experiences of lower‐middle and upper‐middle‐income countries. Included articles largely represented original research (54%), adult cancer populations (61%), and studied patients as the targeted population (51%). More than a quarter of articles focused exclusively on breast cancer (28%). Approximately 30% described factors that influenced decisions to choose between therapies with curative versus non‐curative intent. Of 56 reported factors, 22 novel factors were identified. Socioeconomic status, reimbursement policies/cost of treatment, and treatment and supportive care were the most commonly described factors.

**Conclusions:**

This scoping review expanded upon previously described factors that influence cancer treatment decision‐making in HICs, broadening knowledge to include perspectives of low‐ and middle‐income countries. While global commonalities exist, certain variables influence treatment choices differently or uniquely in different settings. Treatment regimens should further be tailored to local environments with consideration of contextual factors and accessible resources that often impact decision‐making.

## Graphical abstract

Decision‐making is a key part of cancer care and during this process, many factors are considered, related to the attributes of the individual, disease, and context. To develop tools that support decision‐making, understanding which factors are considered is important. In this scoping review, we evaluated existing publications to identify factors influencing treatment decision‐making for children and adults diagnosed with cancer in low‐ and middle‐income countries.

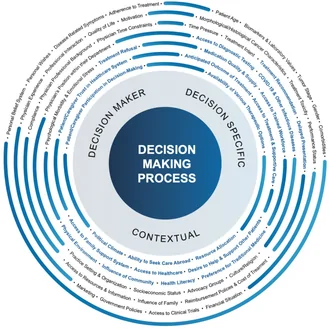

## 1 INTRODUCTION

Decision‐making is an integral component of cancer care, often involving multiple individuals or groups and ideally customized to align with patient and family preferences and goals. Within each unique decision, clinicians, patients, and families must balance nuanced factors related to the attributes of the individual, disease, and context.^1^ Understanding the factors that influence multi‐level decision‐making across different geographies, cultures, and resources is critical to the successful creation and implementation of tools to support decision‐making, including treatment guidelines.

Presently, most studies exploring treatment decision‐making in oncology have been conducted in high‐income countries (HICs),^1^, ^2^, ^3^, ^4^ despite the disproportionate and higher burden of cancer with poorer outcomes in low‐ and middle‐income countries.^5^, ^6^, ^7^ Cancer is a leading cause of mortality worldwide; with strengthening and development of health systems, identification and diagnosis of cancers is expected to increase, particularly in low‐ and middle‐income countries where a majority of cancer deaths already occur.^5^, ^6^, ^8^, ^9^, ^10^ Importantly, decisions around pursuit of treatment with curative versus non‐curative intent often arise in low‐ and middle‐income countries, due in part to unique challenges within healthcare systems in resource‐limited settings.^5^, ^11^, ^12^, ^13^ Treatment with non‐curative intent refers to treatment that will not lead to cure, but rather is focused on improving symptoms, quality of life, or prolonging life. In addition to consideration of the patient's clinical presentation, barriers to treatment with curative intent include inadequate healthcare infrastructure, limited access to care and treatment availability, financial burdens of cancer treatment that drive treatment abandonment, and distrust in the medical system leading to pursuit of traditional medicine.^5^, ^11^, ^12^, ^13^ Despite unique challenges to care, physicians who treat cancer in these settings frequently rely on standardized treatment regimens or protocols to guide therapy for patients; many such regimens center on evidence from therapies developed, delivered, and investigated in HICs.^5^, ^8^, ^14^

To address this limitation, adapted treatment regimens have been developed to stratify cancer‐directed therapy recommendations based on locally available infrastructure and resources.^15^, ^16^, ^17^, ^18^, ^19^, ^20^, ^21^, ^22^, ^23^, ^24^, ^25^, ^26^, ^27^, ^28^, ^29^, ^30^, ^31^, ^32^, ^33^, ^34^, ^35^, ^36^ These regimens typically still focus on treatment with curative intent and are available for select cancer types.^15^, ^16^, ^17^, ^18^, ^19^, ^20^, ^21^, ^22^, ^23^, ^24^, ^25^, ^26^, ^27^, ^28^, ^29^, ^30^, ^31^, ^32^, ^33^, ^34^, ^35^, ^36^ While such adapted regimens are valuable, these guidelines do not consistently meet the needs of local physicians faced with difficult decision‐making scenarios, including lack of guidance surrounding the provision of treatment with non‐curative intent, or account for local resources or contextual factors that may vary and contribute heavily to decision‐making.^8^, ^20^ Understanding and accounting for these factors is crucial for the development of flexible and contextual treatment guidelines reflecting circumstances faced in order to improve outcomes for patients diagnosed with cancer in low‐ and middle‐income countries.

In this scoping review, we evaluated existing literature related to treatment decision‐making for patients diagnosed with cancer in low‐ and middle‐income countries, identifying discrete factors that influenced decision‐making for patients, families and caregivers, healthcare professionals, and community members. We compared these factors with those described in a framework for adult cancer patients developed in a HIC context with the goal of identifying novel factors that influence decision‐making in low‐ and middle‐income country settings in both pediatric and adult oncology.^1^ In addition to this broader scope, we noted factors that influenced decision‐making between treatment with curative versus non‐curative intent.

## 2 METHODS

We used scoping review methodology to identify and describe existing literature on factors influencing decision‐making in low‐ and middle‐income countries for pediatric and adult cancer patients.^37^ A scoping review facilitates the systematic aggregation and synthesis of published knowledge to answer a broad and exploratory research question with the goal of mapping concepts and identifying gaps in research.^38^, ^39^ We purposefully selected this methodology to address an expansive and preliminary research question—*which factors influence cancer treatment decision‐making in low‐ and middle‐income countries*—to build upon an existing HIC framework and recognize research gaps to inform recommendations for future inquiry.^1^, ^38^ This methodology was guided by the Preferred Reporting Items for Systematic Reviews and Meta‐Analysis (PRISMA) Extension for Scoping Reviews to ensure rigorous evaluation and reporting.^40^ The International Prospective Register of Systematic Reviews is not applicable for scoping reviews.^41^ A medical librarian collaborated with researchers to design and apply an advanced Boolean search strategy (Table S1) across PubMed, Embase, Scopus, Global Health, Cumulative Index to Nursing and Allied Health Literature (CINAHL), and WorldWideScience.org databases, inclusive of all article types (e.g., original research, abstracts, reviews, commentaries) from inception through July 2022. Search results were uploaded to Covidence, a web‐based platform for management of systematic and scoping reviews,^42^ and duplicates were identified and removed.

Table S2 presents inclusion and exclusion criteria. Included articles focused on decision‐making by any involved party (patient, family/caregiver, healthcare professional, or community member) during the treatment trajectory for children or adults diagnosed with cancer in low‐ and middle‐income countries. Nine authors (M.S., A.S., P.Y.H.G., S.G., C.D., M.M., A.T., D.F.H., N.M.) were trained to screen titles/abstracts and full texts that met inclusion criteria. Two independent reviewers performed each screening for eligibility with third‐party adjudication of discrepancies (M.S., A.S., E.C.K.).

Two authors (M.S. and A.S.) independently extracted data including study design (qualitative, quantitative, or mixed method), study aim(s), setting, year of publication, patient focus (pediatric or adult), study participants, disease focus, factors influencing treatment decision‐making, and whether the study described how given factors impacted the decision to choose between curative and non‐curative therapies. Third‐party adjudication was performed when necessary (E.C.K.). Results were sorted geographically to include countries defined as low‐income countries (LICs), lower‐middle‐income countries (LMICs), and upper‐middle‐income countries (UMICs) by the World Bank.^43^ If the article did not state a specific country, the region of focus was reported. Articles that reported regional perspectives were included in the analysis but were excluded from categorizations stratified by income level. When articles included the experiences of both low‐ and middle‐income countries and HIC together, we reviewed the article and assessed data specific to the low‐ and middle‐income country context when possible; these articles were categorized by the represented low‐ and middle‐income country.

Deductive and inductive approaches were used to identify and categorize factors influencing treatment decision‐making. A previously published conceptual model of variables that impact decision‐making in oncology^1^ was used as a template to elicit and categorize decision‐making factors identified in this review. In this model, factors were categorized by the decision‐maker (related to the individual decision‐makers' characteristics), the decision (related to the nature of the decision itself), and context (related to the environment within which the decision is being made).^1^ This model was limited by its development in HIC settings; therefore, authors inductively reviewed articles to identify new criteria or factors that did not fit the existing model. New factors were aggregated and organized thematically. Descriptive results are presented but heterogeneity of articles and outcomes precluded formal meta‐analysis.

## 3 RESULTS

**FIGURE 1:**
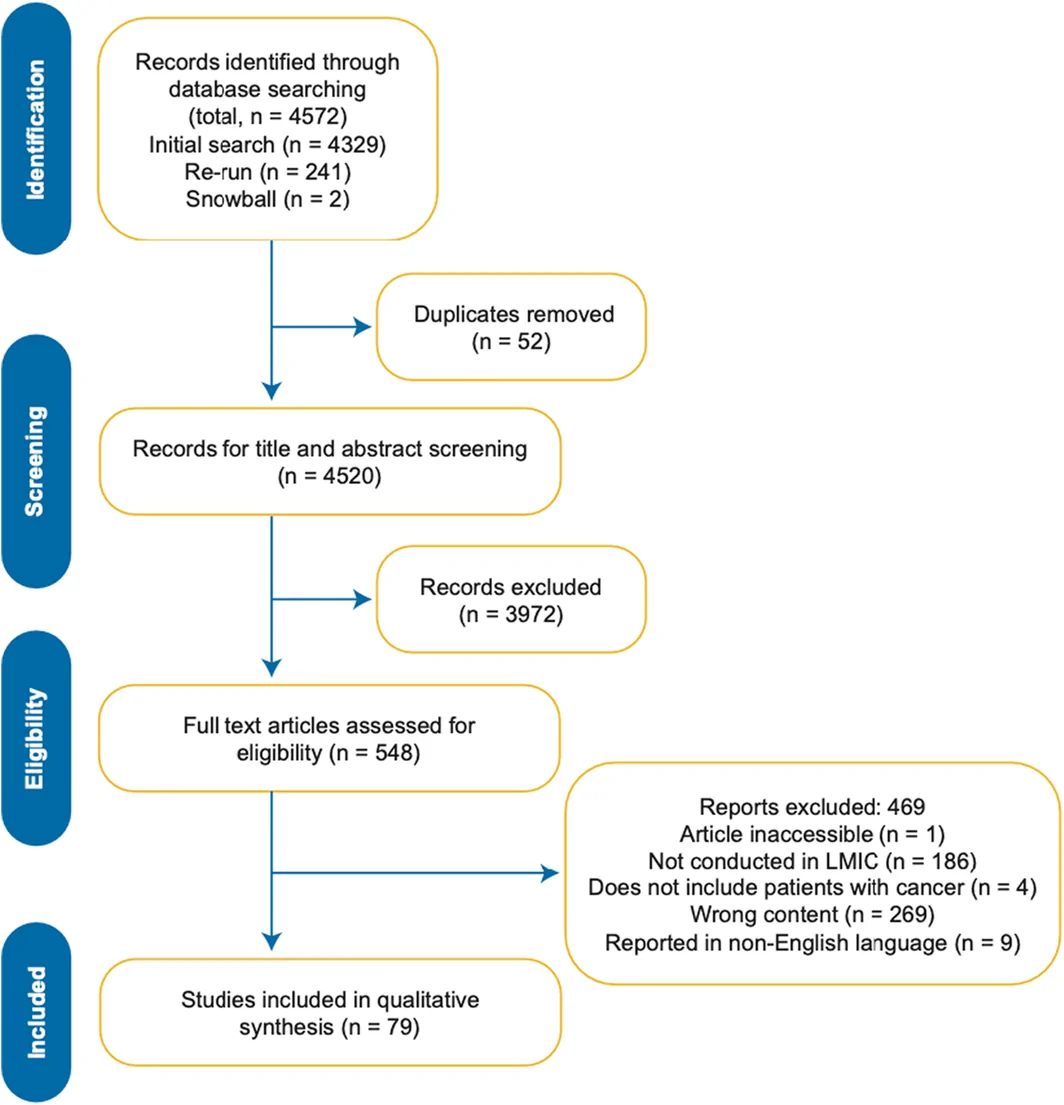
Preferred reporting items for systematic reviews and meta‐analysis (PRISMA) guideline extension for scoping reviews. Flow chart of research results and excluded articles. LMIC indicates low‐ and middle‐income country.

Our search identified a total of 4520 articles with two additional eligible articles added through snowballing (Figure 1). Of these, 79 met inclusion criteria for data abstraction (Figure 1). Twenty‐two countries from six regions (East Asia and Pacific, Europe and Central Asia, Latin America and the Caribbean, Middle East and North Africa, South Asia, Sub‐Saharan Africa) were represented.^43^ Table 1 summarizes the distribution of included articles by income classification, country, and region. The highest proportion of articles were conducted in India (25%) and China (16%), or in countries categorized as LMICs (51%). A single study was reported to have been conducted in a LIC (Uganda). Five articles were not specifically linked to a specific country, instead reporting the experience of a region inclusive of LICs, LMICs, and UMICs.

**TABLE 1:** Number of included articles by World Bank income classification and country or region.

Group by income level	Country	No. of articles (*n*)	%	References
*Low‐income countries*				
	Uganda	1		[96]
Total		1	1	
*Lower‐middle‐income countries*				
	Cameroon	1		[84]
	Egypt	1		[90]
	Ghana	3		[82, 86, 87]
	India	20		[44, 52, 54, 56, 62, 66, 67, 80, 89, 92, 95, 97, 98, 102, 103, 111, 116, 117, 119, 120]
	Indonesia	1		[51]
	Iran	4		[88, 108, 114, 118]
	Lebanon	1		[75]
	Nepal	1		[68]
	Nigeria	3		[63, 112, 121]
	Pakistan	1		[72]
	Philippines	2		[70, 107]
	Tanzania	1		[74]
	Zimbabwe	1		[93]
Total		40	51	
*Upper‐middle‐income countries*				
	Brazil	5		[50, 57, 77, 78, 99]
	China	13		[46, 47, 48, 53, 58, 59, 60, 76, 85, 91, 101, 106, 115]
	Iraq	1		[113]
	Jordan	3		[64, 79, 105]
	Malaysia	5		[46, 49, 71, 73, 83]
	Thailand	1		[100]
	Turkey	4		[55, 61, 69, 81]
	South Africa	1		[122]
Total		33	42	
By Region				
	Central and South America	1		[65]
	Sub‐Saharan Africa	1		[109]
	Multi‐Region	3		[94, 104, 110]
Total		5	6	

Table 2 summarizes the characteristics of included articles. Articles described decision‐making across the cancer treatment continuum and included decision‐making at diagnosis through relapse of disease and transition from treatment with curative intent to non‐curative, comfort focused care. Most included articles presented original research (54%), with application of quantitative and qualitative methods. Sixty‐one percent of articles focused solely on adult cancer populations, and 10% focused on pediatric cancer. Many publications focused on patients (active cancer patients or survivors) as the study population (51%), followed by multidisciplinary healthcare professionals such as physicians, trainees, nurses, social workers, physiotherapists, or pharmacists (21%). Few articles reflected the perspectives of caregivers and community members (e.g., policy makers, religious scholars, lay people). The number of participants ranged from a single patient described in a case report^44^ to study participation ranging from 8^45^ to 2220.^46^ The largest proportion of articles included <50 participants (31%). More than half of articles focused on solid tumors (56%). Notably, more than a quarter of articles focused exclusively on breast cancer (28%). Fourteen percent of articles presented data for more than one cancer type and were listed as “mixed.” Of the publications presenting original research, 44% directly studied decision‐making as a primary aim of the study. Approximately one‐third of articles (30%) described factors that influenced a decision to choose between treatment with curative versus non‐curative intent across the course of cancer treatment.

**TABLE 2:** Characteristics of the articles included in this scoping review.

Characteristic		No. (*n*)	%	Reference
*Article type*				
	Original Research	43	54	[45, 49, 51, 53, 55, 56, 58, 59, 61, 63, 64, 65, 67, 68, 69, 71, 73, 74, 75, 77, 81, 82, 83, 85, 86, 87, 88, 89, 90, 91, 94, 95, 100, 105, 106, 111, 112, 114, 116, 118, 119, 121, 122]
	Abstract	21	27	[46, 47, 48, 52, 60, 62, 70, 76, 78, 84, 92, 93, 96, 97, 99, 101, 102, 103, 113, 115, 117]
	Commentary	6	8	[50, 104, 107, 108, 109, 110]
	Review	5	6	[54, 57, 66, 80, 98]
	Other	4	5	[44, 72, 79, 120]
*Study design*				
	Quantitative	30	38	[46, 47, 53, 55, 56, 58, 59, 60, 61, 62, 64, 65, 68, 69, 71, 78, 83, 90, 95, 96, 97, 99, 100, 103, 105, 111, 113, 114, 115, 117]
	Qualitative	20	25	[45, 49, 51, 63, 67, 72, 73, 74, 81, 82, 85, 86, 87, 89, 91, 112, 118, 119, 121, 122]
	Mixed Methods	7	9	[75, 77, 84, 92, 93, 94, 116]
	Not specified/Not applicable	22	28	[44, 48, 50, 52, 54, 57, 66, 70, 72, 76, 79, 80, 98, 101, 102, 104, 106, 107, 108, 109, 110, 120]
*Patient population focus*				
	Adult	48	61	[44, 45, 46, 47, 48, 49, 51, 53, 55, 56, 58, 59, 60, 61, 62, 63, 64, 69, 70, 71, 72, 73, 75, 80, 82, 83, 85, 86, 87, 90, 91, 94, 95, 96, 99, 100, 101, 103, 104, 105, 107, 108, 115, 116, 118, 119, 120, 121]
	Pediatric	8	10	[65, 67, 81, 84, 88, 93, 106, 110]
	Pediatric & Adult	3	4	[66, 74, 111]
	Not specified/Not applicable	20	25	[50, 52, 54, 57, 68, 76, 77, 78, 79, 89, 92, 97, 98, 102, 109, 112, 113, 114, 117, 122]
*Study population*				
	Patients	40	51	[44, 45, 46, 48, 51, 53, 56, 59, 60, 61, 62, 63, 64, 66, 69, 70, 71, 72, 73, 79, 82, 83, 84, 86, 90, 92, 95, 96, 99, 100, 102, 103, 106, 111, 115, 116, 118, 119, 120, 121]
	Healthcare Professional	17	21	[47, 55, 58, 65, 68, 74, 77, 78, 89, 93, 94, 97, 101, 113, 114, 117, 122]
	Patients + Caregivers	4	5	[75, 85, 88, 91]
	Patients + Healthcare Professionals	2	3	[67, 87]
	Healthcare Professionals + Community Members	2	3	[49, 105]
	Caregivers	1	1	[81]
	Healthcare Professionals + Caregivers	1	1	[112]
	Not specified/Not applicable	12	15	[50, 52, 54, 57, 76, 80, 98, 104, 107, 108, 109, 110]
*Number of participants*				
	<50	25	31	[44, 45, 49, 51, 56, 63, 67, 73, 74, 81, 82, 85, 86, 87, 88, 89, 90, 91, 97, 101, 112, 118, 119, 121, 122]
	50–199	18	23	[55, 60, 61, 62, 64, 68, 71, 75, 77, 90, 92, 96, 99, 100, 102, 111, 114, 117]
	>200	18	23	[46, 48, 53, 58, 59, 69, 70, 83, 84, 94, 95, 103, 105, 106, 113, 115, 116, 120]
	Not specified/Not applicable	18	23	[50, 52, 54, 57, 65, 66, 72, 76, 78, 79, 80, 93, 98, 104, 107, 108, 109, 110]
*Diagnosis type*				
	Solid Tumor	44	56	[44, 45, 46, 48, 49, 50, 53, 56, 59, 60, 61, 62, 63, 64, 66, 69, 70, 71, 72, 73, 77, 78, 80, 82, 83, 85, 86, 87, 90, 91, 92, 94, 95, 97, 99, 100, 101, 104, 105, 108, 116, 120, 121, 122]
	Hematologic	6	8	[47, 58, 96, 98, 103, 106]
	Central Nervous System	1	1	[57]
	Mixed	11	14	[65, 67, 68, 75, 81, 84, 88, 102, 111, 118, 119]
	Not specified/Not applicable	17	21	[51, 52, 54, 55, 74, 76, 79, 89, 93, 107, 109, 110, 112, 113, 114, 115, 117]
*Does the article discuss how factors influence the decision to choose between treatment with curative or non‐curative intent?*				
	Yes	24	30	[46, 47, 50, 55, 58, 74, 77, 79, 80, 86, 89, 91, 93, 94, 95, 96, 98, 99, 102, 104, 106, 109, 111, 113]
	No	55	70	[44, 45, 48, 49, 51, 52, 53, 54, 56, 57, 59, 60, 61, 62, 63, 64, 65, 66, 67, 68, 69, 70, 71, 72, 73, 75, 76, 78, 81, 82, 83, 84, 85, 87, 88, 90, 92, 97, 100, 101, 103, 105, 107, 108, 110, 112, 114, 115, 116, 117, 118, 119, 120, 121, 122]

*Note*: “Other” article type includes letter to editor, case report, perspective, and a poster presentation.

### 3.1 Factors influencing decision‐making

**FIGURE 2:**
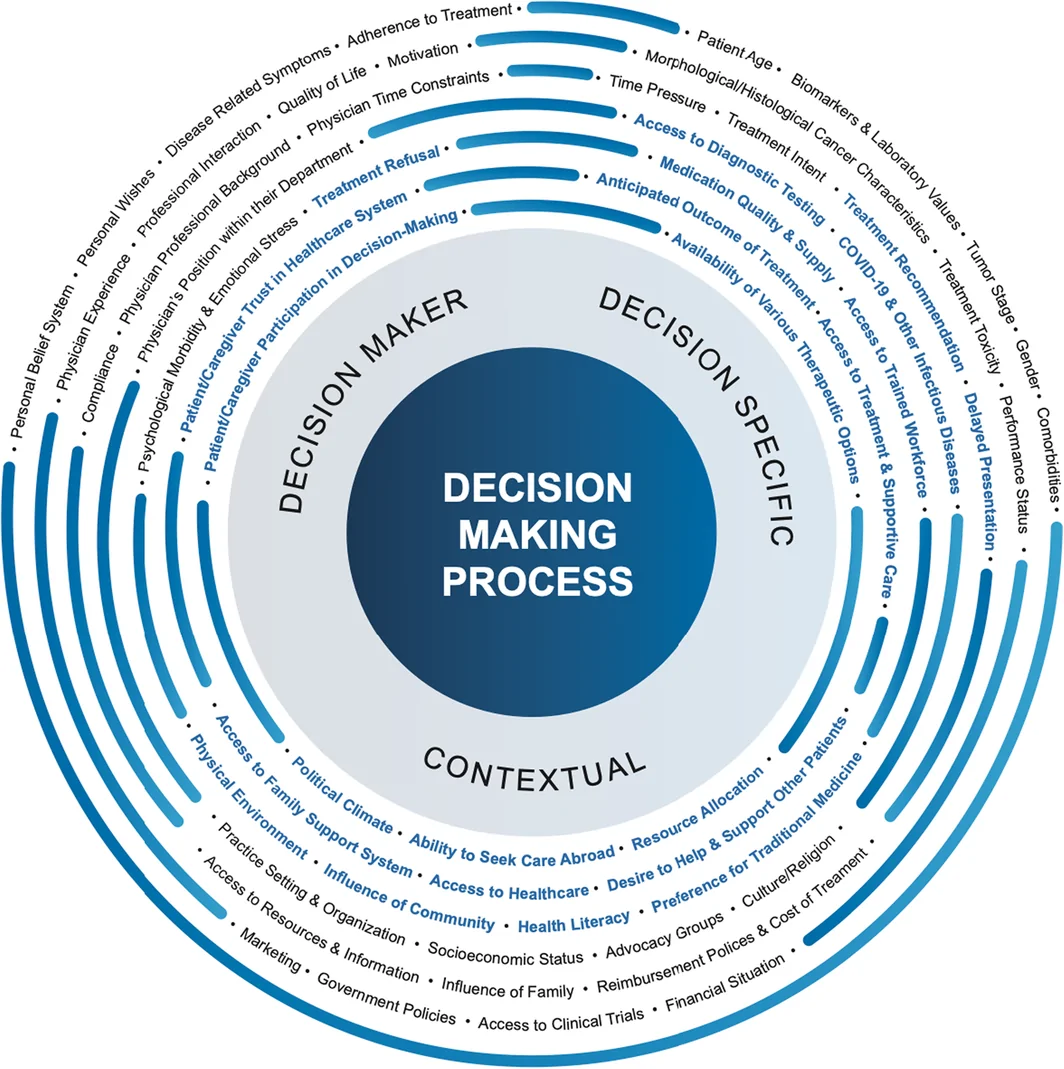
Factors influencing treatment decision‐making in cancer in low‐ and middle‐income countries. A previously published conceptual model of variables that impact decision‐making in oncology was used as a template to elicit categories of decision‐making factors. In this model, factors were categorized by the decision‐maker, decision, and context. This model was limited by its development in high‐income country settings. Articles identified in this review were deductively and inductively reviewed to identify previously described and new criteria or factors that did not fit the existing model. These new factors, highlighted in blue, were aggregated, and organized thematically.

**FIGURE 3:**
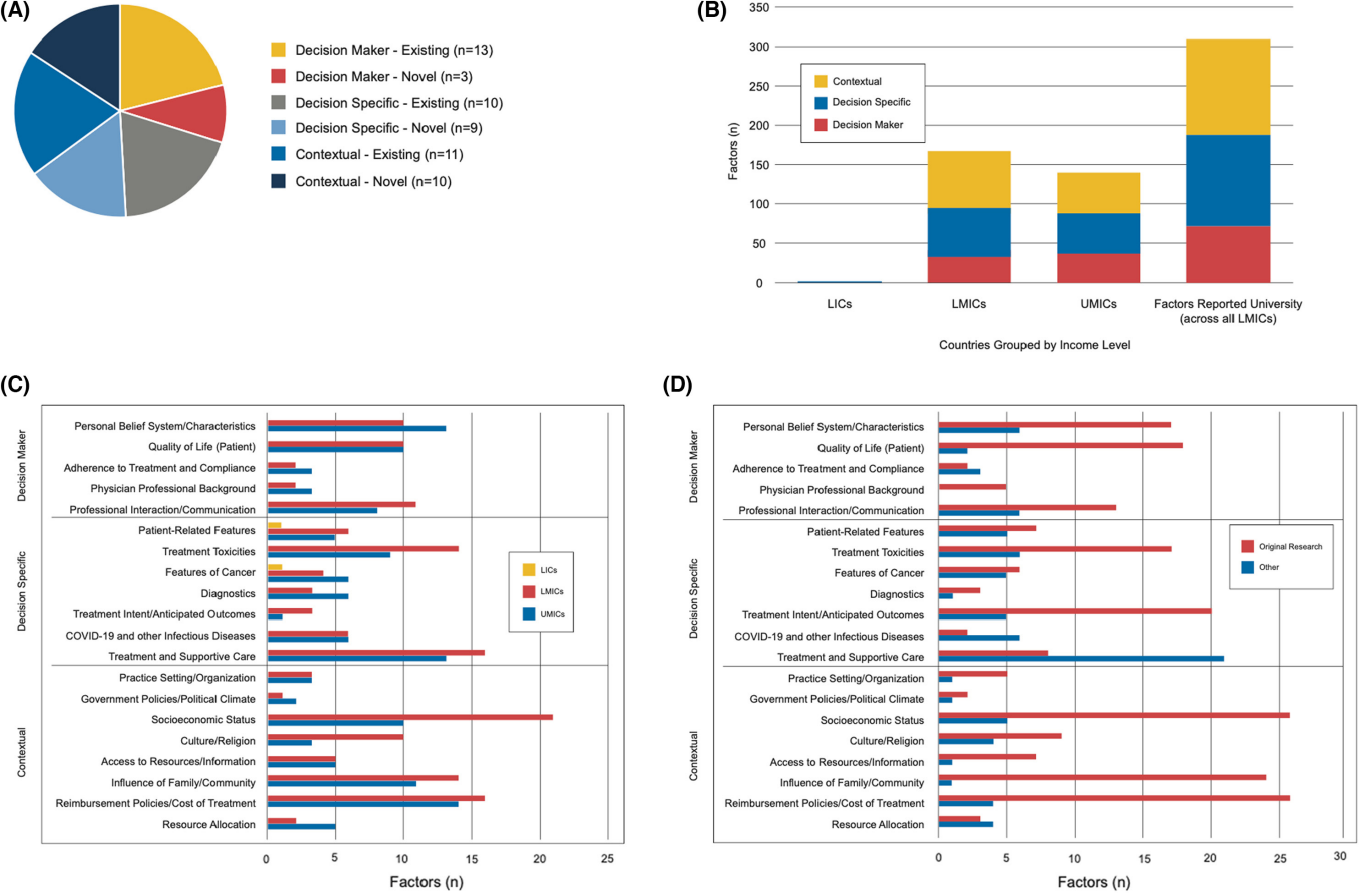
Patterns of factors impacting decision‐making, organized by category and income‐level. Panel A: Summary of factors reported by category, and further sub‐categorized as “existing,” thus identified in the previously published conceptual model related to decision‐making in cancer in high‐income countries or “novel,” meaning that the factor was newly identified as impacting decision‐making in this scoping review. Panel B: Summary of factors identified in this scoping review, listed by category and income level [low‐income countries (LICs), lower‐middle‐income countries (LMICs), upper‐middle‐income countries (UMICs)] as defined by the World Bank.^41^ Panel C: Factors further stratified by category and income level (LICs, LMICs, and UMICs). Few articles have been published to reflect on the LIC approach to decision‐making. Socioeconomic status was the factor most often reported as impacting decision‐making in articles included in this scoping review. Panel D: Factors stratified by category and whether they are described in articles reporting original research.

Identified factors influencing decision‐making for cancer care in low‐ and middle‐income countries are presented in Figure 2. When compared to a conceptual model developed from a HIC perspective,^1^ overlapping similarities were identified along with 22 novel factors (three related to the decision‐maker, nine related to the discrete, disease‐specific decision, and ten related to local context) highlighted in blue in Figure 2 and presented in Figure 3A. These factors were consolidated into 20 unique factor categories (Table 3). Each factor is presented with supporting examples in Table S3. More than one‐third of articles (34%) identified factors across the three domains of decision‐maker, decision itself, and local contexts; 29% identified factors in two of three domains, and 37% identified factors in only one domain (Figure 3B, Table S4). Factor reporting frequency is described in Figure 3C; the most commonly described factors inclusive of all income levels (LICs, LMICs, and UMICs) were related to socioeconomic status, reimbursement policies/cost of treatment, and treatment and supportive care. The single article reporting the experiences from a LIC highlighted the impact of patient‐related and cancer‐specific features. Factors are further delineated based on type of study, original research versus not, in Figure 3D.

**TABLE 3:** Factors affecting treatment decision‐making in pediatric and adult cancer in low‐ and middle‐income countries.

	Decision maker					Decision specific							Contextual							
	Personal belief system/characteristics	Quality of Life	Adherence to treatment and compliance	Physician professional background	Professional interaction/communication	Patient‐related features	Treatment toxicities	Features of cancer	Diagnostics	Treatment intent/anticipated outcomes	COVID‐19 and other infectious diseases	Treatment and supportive care	Practice setting/organization	Government policies/political climate	Socioeconomic status	Culture/religion	Access to resources/information	Influence of family/community	Reimbursement policies/cost of treatment	Resource allocation
Pankaj^44^	•														•	•				
Abdullah^45^																	•	•		
Sun^46^							•			•		•							•	
Wu^47^	•			•																
Zhou^48^	•	•					•			•									•	
Lee^49^	•	•			•		•			•		•						•	•	
Kowalski^50^	•		•		•			•		•		•		•	•				•	•
Deliana^51^	•				•		•	•							•	•		•	•	
Brucker^52^	•															•				
Zhang^53^	•	•					•			•									•	
Doval^54^	•				•	•	•			•		•	•		•	•			•	
Demir Kureci^55^	•		•	•	•					•		•	•	•	•	•		•		
Agrawal^56^	•	•			•		•	•		•		•			•				•	
Batistella^57^	•		•			•	•	•			•									
Wu^58^	•			•									•							
Liu^59^	•							•							•		•		•	
Lin^60^	•																	•		
Gumus^61^	•											•								
Mishra^62^	•	•			•							•			•					
Ogunkorode^63^	•	•		•			•			•					•	•		•	•	
Obeidat^64^	•	•						•		•										
Rosabal‐Obando^65^	•				•				•			•					•			
Mailankody^66^	•	•	•				•					•			•	•			•	
Behan^67^	•				•										•					
Bhandari^68^	•				•				•			•			•		•		•	
Yuksel^69^	•	•								•		•						•		
De Guzman^70^					•		•			•		•			•			•		
Teh^71^		•				•				•		•						•		
Abbasi^72^																	•			
Shariff^73^		•			•					•		•						•		
Harris^74^							•			•		•			•	•	•	•	•	
Skelton^75^									•			•		•	•			•	•	
Tang^76^		•			•	•		•										•	•	
Pereira de Veiga^77^								•	•			•	•		•		•		•	•
Amaro^78^												•					•			
Al‐Tabba^79^					•		•			•	•									•
Bhatla^80^						•	•	•	•	•	•	•					•			•
Kilicarslan‐Toruner^81^		•			•							•			•		•		•	
Aziato^82^		•		•								•				•		•		
Muhamad^83^												•						•		
Kouya^84^					•															
Wang^85^					•		•								•					
Salisu^86^					•							•	•		•	•		•		
Agyemang^87^		•	•		•		•					•			•	•			•	
Ahmadnia^88^					•							•			•					
Gielen^89^		•				•	•			•								•	•	
El‐Hadidy^90^		•																		
Gong^91^		•								•					•	•		•	•	
Ramakrishnan^92^		•																•	•	
Salek^93^		•					•			•										
Hurdle^94^				•		•						•				•			•	
Baijal^95^						•														
Menon^96^						•		•												
Pawar^97^						•														
Jain^98^						•	•	•			•	•			•		•			•
Peruzzo^99^						•														
Ngorsuraches^100^						•	•												•	
Zhou^101^							•			•					•				•	
Ramesh^102^							•			•	•	•								
Malhotra^103^							•			•		•			•				•	
Del Pilar Estevez‐Diz^104^								•		•	•	•		•						
Yousef^105^												•								•
Hong^106^										•					•				•	
Mendoza^107^										•	•									
Siavashpour^108^											•									
Vanderpuye^109^											•									
Pritchard‐Jones^110^											•									
Nair^111^											•									
Agom^112^													•					•	•	
Shelal^113^																				•
Daroudi^114^																			•	
Li^115^															•				•	
Alexander^116^															•			•	•	
Bhattacharya^117^																			•	
Soltani^118^															•					
Datta^119^															•			•		
Alexander^120^															•			•		
Olasehinde^121^																		•		
Brown^122^																•		•		

*Note*: Factors are categorized as related to the decision maker, decision, and context. Articles shaded in gray reflect those reporting original research (inclusive of abstracts and a poster presentation presenting primary data).

### 3.2 Factors related to the decision‐maker

Factors related to the individual's belief system (separate from culture or religion) or characteristics of the involved individual were described in 30% of articles.^44^, ^47^, ^48^, ^49^, ^50^, ^51^, ^52^, ^53^, ^54^, ^55^, ^56^, ^57^, ^58^, ^59^, ^60^, ^61^, ^62^, ^63^, ^64^, ^65^, ^66^, ^67^, ^68^, ^69^ Patients described factors that motivated their pursuit of treatment, including desire for cure,^70^ hope for recovery,^51^, ^69^ a wish to live,^63^ and a fear of recurrence.^46^, ^53^, ^56^, ^62^, ^69^, ^71^ The personality traits of physicians were also described as factors influencing treatment decision‐making,^47^, ^58^ including an unwillingness to change one's standard clinical practice.^68^

Factors related to professional interaction and communication were described in 25% of articles.^45^, ^46^, ^49^, ^50^, ^51^, ^54^, ^55^, ^56^, ^61^, ^62^, ^65^, ^66^, ^67^, ^68^, ^69^, ^70^, ^71^, ^72^, ^73^, ^74^, ^75^, ^76^, ^77^, ^78^, ^79^, ^80^, ^81^, ^82^, ^83^, ^84^, ^85^, ^86^, ^87^, ^88^ Factors related to professional interaction and communication in articles that focused on the perspective of patients and families included the invitation to participate in decision‐making,^54^, ^62^, ^65^, ^70^, ^88^ trust,^70^, ^73^, ^81^ or distrust^49^, ^51^, ^86^ in their healthcare providers, variable quality of patient‐healthcare team communication,^79^, ^86^, ^87^ including disempowerment of patients, lack of patient understanding of therapy or therapeutic options, and fear of raising questions risking the potential for inferior care.^87^ Additional factors included deliberate miscommunication to convince a patient to accept care,^87^ time constraints of the physician,^49^, ^50^, ^68^ and language barriers that prevented the effective description of cancer and cancer therapies with patients and families.^87^

Approximately one‐quarter of articles (27%) described factors related to quality of life.^48^, ^49^, ^53^, ^56^, ^62^, ^63^, ^64^, ^66^, ^69^, ^71^, ^73^, ^76^, ^81^, ^82^, ^87^, ^89^, ^90^, ^91^, ^92^, ^93^ These factors were described broadly, such as “wanting to live a longer, healthier, enjoyable life” or spending time with loved ones and traveling.^73^ Quality of life was also described in relation to cancer treatment, including preference for the mode of medication administration,^48^ tolerance of side effects,^49^ impact on functional status or risk for disfigurement,^53^, ^66^, ^69^, ^92^ potential for suffering,^89^, ^93^ impact on family planning,^53^, ^55^, ^87^ fear of undergoing treatment,^63^, ^64^, ^69^, ^71^ and preference to minimize length of treatment.^64^ In the setting of breast cancer, the quality of life factor encompassed perception of impact on the female body shape,^53^, ^56^, ^62^, ^64^, ^71^, ^82^ on marriage and fertility,^53^, ^69^, ^71^, ^82^, ^87^ the psychological co‐morbidity of the patient and/or their husband,^64^, ^90^ and preference to avoid second surgery.^69^, ^71^

Additional themes identified included the influence of the professional background of the physician,^47^, ^55^, ^58^, ^63^, ^82^, ^94^ such as their clinical experience^55^, ^58^, ^94^ and prior training.^58^, ^94^ Further, physician perceptions of risk of non‐compliance, treatment refusal, or abandonment were factors that influenced treatment decision‐making.^50^, ^55^, ^57^, ^66^, ^87^

### 3.3 Factors related to the decision

Patient characteristics such as age, gender, comorbidities, and performance status influenced treatment decision‐making.^54^, ^57^, ^71^, ^76^, ^80^, ^89^, ^94^, ^95^, ^96^, ^97^, ^98^, ^99^, ^100^ One study described gender bias in the treatment of osteosarcoma and the neglect of female patients.^66^ Other factors included treatment toxicities,^46^, ^48^, ^49^, ^51^, ^53^, ^54^, ^56^, ^57^, ^63^, ^66^, ^70^, ^74^, ^79^, ^80^, ^85^, ^87^, ^89^, ^93^, ^98^, ^100^, ^101^, ^102^, ^103^ specific disease features,^50^, ^51^, ^56^, ^57^, ^59^, ^64^, ^76^, ^77^, ^80^, ^96^, ^98^, ^104^ the results of diagnostic workup,^65^, ^75^, ^77^, ^80^ and access to cancer‐directed treatment and supportive care resources and personnel.^46^, ^49^, ^50^, ^51^, ^54^, ^55^, ^56^, ^61^, ^62^, ^65^, ^66^, ^68^, ^70^, ^73^, ^74^, ^75^, ^77^, ^78^, ^80^, ^81^, ^82^, ^83^, ^84^, ^86^, ^87^, ^88^, ^94^, ^98^, ^102^, ^103^, ^104^, ^105^

Treatment intent and the anticipated outcome of treatment were described as factors influencing treatment decision‐making in 34% of articles.^46^, ^49^, ^50^, ^53^, ^54^, ^55^, ^56^, ^63^, ^64^, ^69^, ^70^, ^71^, ^73^, ^74^, ^79^, ^80^, ^91^, ^93^, ^101^, ^102^, ^103^, ^104^, ^106^, ^107^ For example, estimated prognosis,^70^, ^89^ likelihood of benefit from treatment,^70^, ^74^, ^89^, ^107^ curability,^74^, ^93^, ^102^, ^106^ extension of life,^64^, ^102^ survival at the expense of quality of life,^91^, ^93^ or a goal to prevent recurrence^64^, ^73^ were described as salient factors.

Ten articles (13%) in this review described the impact of COVID‐19 on treatment decision‐making for cancer in LMICs.^57^, ^79^, ^80^, ^98^, ^102^, ^104^, ^107^, ^108^, ^109^, ^110^ Otherwise, infectious disease was considered in only one other article related to tuberculosis.^111^

### 3.4 Contextual factors

Contextual factors describe a broad category of variables occurring in the environment within which a decision is made.^1^ For example, decision‐making may be influenced by a practice setting or organization,^54^, ^55^, ^58^, ^77^, ^86^, ^112^ hospital funding,^77^ protocols, or bureaucracies.^86^ Contextual factors also may include government policies, such as visa regulations^75^ or national policies that impact access to resources or personnel,^55^ or the political climate, including war.^50^, ^55^, ^75^, ^104^ Physicians report variable access to resources and information to help guide decision‐making, including treatment guidelines or protocols,^68^, ^77^, ^80^ multidisciplinary tumor boards^65^, ^68^, ^72^, ^77^, ^78^, ^80^ or knowledgeable colleagues.^68^, ^74^ A country's economic status may further limit resource allocation, directly and indirectly impacting treatment decision‐making.^50^, ^77^, ^79^, ^80^, ^98^, ^105^, ^113^

More than one‐third of articles (40%) discussed the impact of treatment cost from a health system perspective, including access to reimbursement.^46^, ^48^, ^49^, ^50^, ^51^, ^53^, ^54^, ^56^, ^59^, ^63^, ^66^, ^68^, ^74^, ^75^, ^76^, ^77^, ^81^, ^87^, ^89^, ^91^, ^92^, ^94^, ^100^, ^101^, ^103^, ^106^, ^112^, ^114^, ^115^, ^116^, ^117^ These factors include cost and affordability of treatment,^68^, ^87^, ^103^, ^114^, ^118^ insurance access,^50^, ^59^, ^66^, ^94^, ^115^ and a clinic's goal to achieve the best clinical response at the lowest cost.^50^, ^91^

Similarly, the impact of socioeconomic factors on treatment decision‐making was discussed in 41% of articles.^44^, ^50^, ^51^, ^54^, ^55^, ^56^, ^59^, ^62^, ^63^, ^66^, ^67^, ^68^, ^70^, ^73^, ^74^, ^75^, ^77^, ^81^, ^85^, ^86^, ^87^, ^88^, ^91^, ^98^, ^101^, ^103^, ^106^, ^115^, ^116^, ^118^, ^119^, ^120^ This category included the financial capabilities of a patient or family/caregivers to pay for cancer treatment,^62^, ^63^, ^66^, ^74^, ^76^, ^85^, ^87^, ^88^, ^101^, ^103^, ^115^, ^116^, ^118^, ^119^, ^120^ the economic burden of treatment on a household,^74^, ^106^ or the sequelae of poverty.^51^, ^74^ Access to healthcare was described,^70^, ^75^, ^86^ relative to local violence,^75^ geographic restrictions,^50^ transportation challenges including cost and risk associated with travel,^66^, ^74^ and the ability to seek care abroad.^51^ Families also had to consider the costs and logistics of transporting the body of a family member after death.^74^

Family members and the local community were described as a factor impacting treatment decision‐making in one‐third (32%) of articles.^45^, ^49^, ^50^, ^51^, ^55^, ^60^, ^63^, ^69^, ^70^, ^71^, ^73^, ^74^, ^75^, ^76^, ^82^, ^83^, ^86^, ^89^, ^91^, ^92^, ^112^, ^116^, ^119^, ^120^, ^121^, ^122^ Family influences were variable and included assessment that a patient was too weak to undergo treatment^92^ or, conversely, referenced a familial obligation to treat a patient with curative intent.^91^ The impact of a patient being separated from the family was another salient factor influencing treatment decision‐making,^74^ as well as the impact of the patient's role and responsibilities in the family^51^ and the need for support from one's family and community.^63^

Health literacy was also described as an influencing factor, including patient and family perceptions of cancer and associated stigma.^44^, ^54^, ^55^, ^56^, ^62^, ^63^, ^87^, ^92^, ^120^ The influence of the general perception of disease and its treatment in the community was reported.^51^, ^86^ Patients and their families/caregivers occasionally sought resources outside of their medical teams to help guide decision‐making, including social media or cancer survivors.^59^, ^81^ Specific to breast cancer, the opinion of a male family member in treatment decision‐making was described in seven articles.^56^, ^60^, ^62^, ^71^, ^86^, ^116^, ^120^ The influence of culture or religion in decision‐making was reported in 13 articles.^44^, ^50^, ^51^, ^52^, ^54^, ^55^, ^63^, ^66^, ^82^, ^86^, ^87^, ^91^, ^94^, ^122^ Patient or family preferences for engagement with a traditional healer or alternative therapy further influenced treatment decision‐making.^51^, ^66^, ^74^, ^82^, ^86^ Other factors included myths, superstitions, stigma related to cancer diagnosis,^44^, ^63^, ^66^, ^86^, ^87^, ^122^ and perception of destiny or fate.^86^ The need to consult a cultural or spiritual leader prior to initiating therapy was also described.^122^

### 3.5 Factors influencing decision‐making when choosing between treatment with curative and non‐curative intent

Thirty percent of articles discussed factors influencing decision‐making when choosing between treatment with curative and non‐curative intent across the trajectory of cancer treatment, considering patient (e.g., performance status), disease (e.g., metastases), or contextual factors (e.g., financial barriers, limited resources, COVID‐19 pandemic).^46^, ^47^, ^50^, ^55^, ^58^, ^74^, ^77^, ^79^, ^80^, ^86^, ^89^, ^91^, ^93^, ^94^, ^95^, ^96^, ^98^, ^99^, ^102^, ^104^, ^106^, ^109^, ^111^, ^113^

Several articles highlighted decision‐making from the perspective of the patient and family.^46^, ^86^, ^91^, ^95^, ^106^ Some families declined therapy after losing confidence in the curability of a child's disease, for example at time of relapse.^106^ Patients in Ghana diagnosed with breast cancer reported that distrust in the healthcare system and their religious and spiritual beliefs led them to reject physician's recommendations and turn to religious leaders and traditional healers.^86^

The decision‐making process in this circumstance was also reported in articles from the physician or healthcare system perspective.^47^, ^50^, ^55^, ^58^, ^74^, ^77^, ^79^, ^80^, ^89^, ^93^, ^94^, ^98^, ^102^, ^104^, ^109^, ^113^ Financial barriers were also described to impact decision‐making.^50^, ^74^, ^77^, ^89^, ^94^, ^106^, ^109^ Several articles described physician recommendations for or against intensive therapies^47^, ^55^, ^74^; for example, given the high cost of treatment and likely ineffective outcomes, physicians in Tanzania often did not recommend intensive treatment for patients with a cancer diagnosis that could not be cured in their setting, instead recommending palliative care to avoid financial devastation of the family.^74^ At times, these treatment decisions were made unilaterally by the physician, without patient or family involvement.^74^ Some clinicians considered it to be irresponsible to offer intensive treatment without realistic chance of cure, with the potential for adverse outcomes for the patient and family members.^74^

Several articles discussed the impact of the COVID‐19 pandemic on decision‐making and recommendations for treatment with curative versus non‐curative intent. These decisions were influenced by available resources and personnel, risks of delaying treatment, estimated prognosis, patient's clinical condition, risk of adverse patient outcome such as disease progression, myelosuppression, or psychological distress, and local incidence of the virus.^80^, ^98^, ^102^, ^104^, ^109^ Multiple articles recommended postponement of therapies with palliative intent to minimize patient volume within the medical center, focusing rather on those who had chance of cure.^80^, ^109^ Separately, infectious complications were reported in one study, in which a patient's lack of response to treatment of tuberculosis subsequently influenced the decision to forgo further cancer‐directed therapy aimed at cure.^111^

## 4 DISCUSSION

In this scoping review, we sought to improve understanding of the complexities of decision‐making in adult and pediatric cancer care in low‐ and middle‐income countries. Of 56 reported factors influencing decision‐making for cancer patients, 34 were similar in low‐ and middle‐income countries when compared to an existing framework proposed for adult oncology in HICs^1^; importantly, we also identified 22 novel factors across all three categories specific to low‐ and middle‐income countries. Certain factors, including socioeconomic status (e.g., financial circumstances of the family or their access to healthcare), reimbursement policies/cost of treatment, access to treatment and supportive care (e.g., access to high‐quality medications, required treatment modalities to meet clinical needs of patients or trained workforce), and treatment intent/anticipated outcomes, were described more commonly across countries included in this analysis, underscoring potential areas of further inquiry in future investigation of strategies to improve treatment decision‐making experiences and outcomes for cancer patients in these settings.

Certain factors have been described in both HICs and low‐ and middle‐income countries, yet we hypothesize that the magnitude or scale at which some factors influence decision‐making in low‐ and middle‐income country settings may be greater compared to HICs. For example, physician time constraints or access to diagnostics required to confirm a diagnosis or sufficiently stage disease may influence treatment decision‐making globally; however, these factors may have a different or greater impact in low‐ and middle‐income countries compared to HICs due to heightened limitations in the healthcare workforce and more expansive restrictions in access to resources needed to provide high quality cancer care.^5^ Further, while screening programs and referral networks in HICs generally allow for the early identification of cancer, advanced cancer at the time of diagnosis occurs commonly in low‐ and middle‐income countries, amplifying treatment decision‐making challenges.^5^, ^10^

Notably, novel factors inherent to low‐ and middle‐income country settings were identified in this review, such as the impact of political instability or war, the influence of traditional medicine, and constrained access to healthcare personnel and resource allocation. Better understanding of how these factors influence treatment decision‐making across the illness course should inform global efforts to enhance algorithms that guide and support cancer‐directed therapy in low‐ and middle‐income countries in an inclusive and comprehensive manner, reflective of local realities. New factors identified in this review may also impact decision‐making in HICs, potentially strengthening the original conceptual framework.^1^

Importantly, more than a quarter of identified articles focused exclusively on breast cancer, a leading cause of global cancer incidence.^6^ Future opportunities exist to explore factors that influence treatment decision‐making across various cancer types. Similarly, more than half of the represented countries were described in a single article, and the vast majority were presented in five or fewer articles. We found only one study from a designated LIC (Uganda), acutely highlighting the need for development of research partnerships and infrastructure to encourage and facilitate improved reporting of experiences in LICs.^123^

Lastly, decision‐making around delivering therapy with curative versus non‐curative intent is a critical area for future exploration, with particular need in low‐ and middle‐income countries where patients frequently present with advanced or widely metastatic disease at the time of diagnosis.^5^, ^10^ This review provides insights into challenges around navigating curative versus non‐curative treatment decision‐making with high relevance to low‐ and middle‐income countries, including the influence of traditional healers, high cost of treatment preventing the pursuit of treatment with curative intent, varying priorities of the healthcare systems and resource allocation, and the impact of paternalistic decision‐making by clinicians. Of the 24 articles that discussed factors influencing the decision to pursue treatment with curative versus non‐curative intent, none reflected the perspective of the community and only four reflected the perspective of the patient and family/caregivers. Collectively, these findings reveal a critical need and opportunity for future work to explore decision‐making in these circumstances through the lens of all involved parties.

This scoping review had several important limitations. The categorization of these and previously described factors is subjective, and while a factor may have been assigned to one category, it may also impact another due to the nuanced and complex process of decision‐making in cancer care. The organization of results in this review are intended to comprehensively identify and map factors impacting decision‐making in low‐ and middle‐income countries and identify gaps in the literature rather than synthesize the available evidence. Additional models of factors or criteria considered in decision‐making have been conceptualized, such as the framework proposed by Iseli et al.^124^ to identify factors in decision‐making that may not be considered in each clinical encounter. This model considers primarily criteria related to the cancer staging, available diagnostics, patient comorbidities, available treatment, performance scores, treatment access, and response, as well as broad characteristics of the patient and caretaker.^124^ The authors highlight the need to include additional factors in national treatment guidelines and acknowledge the limitation that psychosocial factors were not considered. Arguably, there are further limitations of this model, as it fails to consider additional, nuanced factors in decision‐making, particularly related to context as identified in this review. We hope that our review can inform future research and the development of conceptual frameworks that directly support and reflect decision‐making in low‐ and middle‐income countries.

Moreover, most eligible articles focused on the adult population and represented a limited number of low‐ and middle‐income countries, precluding our ability to generalize findings, in particular to pediatric populations and LIC contexts. Even when a country was represented in the literature, it often had few articles reporting decision‐making experiences in local contexts, and we must be careful not to generalize or over‐interpret the data. Future work should leverage qualitative methodology to characterize nuances within and between specific contexts, and opportunities to gain qualitative research skills should be provided to interested clinicians and researchers in low‐ and middle‐income countries to lead studies in their native languages that describe local realities and barriers. Due to logistical constraints, this review only included articles published in English, resulting in the exclusion of nine publications in other languages; this is a limitation that necessitates attention in future work. Many included articles did not present original research and required descriptive synthesis; this precluded quantitative reporting of aggregated data or meta‐analysis and further highlights the need for supporting future rigorous investigation. Our search strategy also may not have captured all relevant articles. Finally, the framework initially used to categorize decision‐making factors was based off a pre‐existing conceptual model developed in HICs, which may represent a limited template; for this reason, we conducted an inductive review of content to derive novel factors to expand the original framework and include the low‐ and middle‐income country perspective.

In summary, this scoping review expanded upon previously described factors that influence cancer treatment decision‐making, broadening knowledge to include perspectives from low‐ and middle‐income countries. While global commonalities in treatment decision‐making exist, some variables impact treatment choices differently or uniquely in these settings. As data and research efforts in low‐ and middle‐income countries expand, treatment regimens should be tailored utilizing HIC evidence to these local settings with consideration of specific contextual factors and accessible resources that often impact decision‐making. Clinicians and researchers in these countries should receive funding and support to explore unique factors that influence or modify treatment decision‐making in their respective communities from the viewpoints of all involved parties. This knowledge has the potential to help shape and improve cancer care delivery in these settings, optimizing the potential for curative outcomes, while also dedicating support to those diagnosed with incurable or advanced disease. Understanding the unique factors that influence treatment decision‐making in low‐ and middle‐income countries becomes increasingly important as clinicians and researchers strive to improve global cancer care and develop and disseminate therapy guidelines and practical interventions to improve outcomes.

## AUTHOR CONTRIBUTIONS

**Marta Salek:** Conceptualization (lead); data curation (lead); formal analysis (lead); investigation (lead); methodology (lead); project administration (lead); writing – original draft (lead); writing – review and editing (lead). **Allison Silverstein:** Formal analysis (lead); investigation (lead); methodology (lead); project administration (equal); validation (lead); visualization (lead); writing – original draft (lead); writing – review and editing (lead). **Alyssa Tilly:** Formal analysis (equal); writing – review and editing (equal). **Pascale Yola Gassant:** Formal analysis (equal); writing – review and editing (equal). **Sanjeeva Gunasekera:** Formal analysis (equal); writing – review and editing (equal). **Diriba Fufa Hordofa:** Formal analysis (equal); writing – review and editing (equal). **Donna Hesson:** Data curation (equal); writing – review and editing (equal). **Caitlyn Duffy:** Formal analysis (equal); writing – review and editing (equal). **Nauman Malik:** Formal analysis (equal); writing – review and editing (equal). **Michael McNeil:** Formal analysis (equal); writing – review and editing (equal). **Lisa M. Force:** Formal analysis (equal); writing – review and editing (equal). **Nickhill Bhakta:** Conceptualization (equal); formal analysis (equal); methodology (equal); supervision (equal); writing – review and editing (equal). **Danielle Rodin:** Formal analysis (equal); writing – review and editing (equal). **Erica C. Kaye:** Conceptualization (lead); data curation (lead); formal analysis (lead); investigation (lead); methodology (lead); resources (lead); supervision (lead); validation (lead); visualization (lead); writing – original draft (lead); writing – review and editing (lead).

## FUNDING INFORMATION

This work was supported in part by ALSAC (American Lebanese Syrian Associated Charities). Additionally, Dr. Kaye receives salary support from the National Cancer Institute (K08CA266935).

## CONFLICT OF INTEREST STATEMENT

No authors have conflicts of interest related to this manuscript to disclose.

## Supporting information


**Data S1.** Supporting Information.

## References

[1] Glatzer M , Panje CM , Siren C , et al. Decision making criteria in oncology. Oncology. 2020;98:370‐378. 30227426 10.1159/000492272

[2] Whitney SN , Ethier AM , Fruge E , et al. Decision making in pediatric oncology: who should take the lead? The decisional priority in pediatric oncology model. J Clin Oncol. 2006;24:160‐165. 16382126 10.1200/JCO.2005.01.8390

[3] Mack JW , Cronin AM , Kang TI . Decisional regret among parents of children with cancer. J Clin Oncol. 2016;34:4023‐4029. 27621402 10.1200/JCO.2016.69.1634

[4] Snaman JM , Helton G , Holder RL , Revette A , Baker JN , Wolfe J . Identification of adolescents and young adults' preferences and priorities for future cancer treatment using a novel decision‐making tool. Pediatr Blood Cancer. 2021;68:e28755. 33017087 10.1002/pbc.28755

[5] Atun R , Bhakta N , Denburg A , et al. Sustainable care for children with cancer: a lancet oncology commission. Lancet Oncol. 2020;21:e185‐e224. 32240612 10.1016/S1470-2045(20)30022-X

[6] Sung H , Ferlay J , Siegel RL , et al. Global cancer statistics 2020: GLOBOCAN estimates of incidence and mortality worldwide for 36 cancers in 185 countries. CA Cancer J Clin. 2021;71:209‐249. 33538338 10.3322/caac.21660

[7] Bhakta N , Force LM , Allemani C , et al. Childhood cancer burden: a review of global estimates. Lancet Oncol. 2019;20:e42‐e53. 30614477 10.1016/S1470-2045(18)30761-7

[8] Carlson RW , Scavone JL , Koh W , et al. NCCN framework for resource stratification: a framework for providing and improving global quality oncology care. J Natl Compr Canc Netw. 2016;14:961‐969. 27496112 10.6004/jnccn.2016.0103

[9] Rodriguez‐Galindo C , Friedrich P , Alcasabas P , et al. Toward the cure of all children with cancer through collaborative efforts: pediatric oncology as a global challenge. J Clin Oncol. 2015;33:3065‐3073. 26304881 10.1200/JCO.2014.60.6376 PMC4979198

[10] Hannon B , Zimmermann C , Knaul FM , Powell RA , Mwangi‐Powell FN , Rodin G . Provision of palliative care in low‐ and middle‐income countries: overcoming obstacles for effective treatment delivery. J Clin Oncol. 2016;34:62‐68. 26578612 10.1200/JCO.2015.62.1615

[11] Knaul FM , Gralow JR , Atun R , Bhadelia A , Fineberg HV , Sen A . Closing the Cancer Divide: An Equity Imperative. Harvard University Press; 2012.

[12] Kaasa S , Loge JH , Aapro M , et al. Integration of oncology and palliative care: a lancet oncology commission. Lancet Oncol. 2018;19:e588‐e653. 30344075 10.1016/S1470-2045(18)30415-7

[13] Israels T , Afungchwi GM , Chagaluka G , et al. Early death and treatment‐related mortality: a report from SUCCOUR—supportive Care for Children with cancer in Africa. Pediatr Blood Cancer. 2021;68:e29230. 34245228 10.1002/pbc.29230

[14] Hunter N , Dempsey N , Tbaishat F , Jahanzeb M , al‐Sukhun S , Gralow JR . Resource‐stratified guideline‐based cancer care should be a priority: historical context and examples of success. ASCO Educ Book. 2020;40:217‐226. 10.1200/EDBK_279693 32223670

[15] Hunger SP , Sung L , Howard SC . Treatment strategies and regimens of graduated intensity for childhood acute lymphoblastic leukemia in low‐income countries: a proposal. Pediatr Blood Cancer. 2009;52:559‐565. 19127567 10.1002/pbc.21889

[16] Bansal D , Davidson A , Supriyadi E , Njuguna F , Ribeiro RC , Kaspers GJL . SIOP PODC adapted risk stratification and treatment guidelines: recommendations for acute myeloid leukemia in resource‐limited settings. Pediatr Blood Cancer. 2019;e28087. 31774234 10.1002/pbc.28087

[17] Chantada G , Luna‐Fineman S , Sitorus RS , et al. SIOP‐PODC recommendations for graduated‐intensity treatment of retinoblastoma in developing countries. Pediatr Blood Cancer. 2013;60:719‐727. 23335388 10.1002/pbc.24468

[18] Hesseling P , Israels T , Harif M , Chantada G , Molyneux E , Pediatric Oncology in Developing Countries . Practical recommendations for the management of children with endemic Burkitt lymphoma (BL) in a resource limited setting. Pediatr Blood Cancer. 2013;60:357‐362. 23192950 10.1002/pbc.24407

[19] Hessissen L , Parkes J , Amayiri N , et al. SIOP PODC adapted treatment guidelines for low grade gliomas in low and middle income settings. Pediatr Blood Cancer. 2017;64(Suppl 5):S1‐S15. 10.1002/pbc.26737 29297618

[20] Howard SC , Davidson A , Luna‐Fineman S , et al. A framework to develop adapted treatment regimens to manage pediatric cancer in low‐ and middle‐income countries: the pediatric oncology in developing countries (PODC) Committee of the International Pediatric Oncology Society (SIOP). Pediatr Blood Cancer. 2017;64(Suppl 5):S1‐S18. 10.1002/pbc.26879 29297619

[21] Israels T , Moreira C , Scanlan T , et al. SIOP PODC: clinical guidelines for the management of children with Wilms tumour in a low income setting. Pediatr Blood Cancer. 2013;60:5‐11. 23015404 10.1002/pbc.24321

[22] Israels T , Renner L , Hendricks M , et al. SIOP PODC: recommendations for supportive care of children with cancer in a low‐income setting. Pediatr Blood Cancer. 2013;60:899‐904. 23441092 10.1002/pbc.24501

[23] Ladas EJ , Arora B , Howard SC , Rogers PC , Mosby TT , Barr RD . A framework for adapted nutritional therapy for children with cancer in low‐ and middle‐income countries: a report from the SIOP PODC nutrition working group. Pediatr Blood Cancer. 2016;63:1339‐1348. 27082376 10.1002/pbc.26016

[24] Molyneux E , Davidson A , Orem J , et al. The management of children with Kaposi sarcoma in resource limited settings. Pediatr Blood Cancer. 2013;60:538‐542. 23255282 10.1002/pbc.24408

[25] Parikh NS , Howard SC , Chantada G , et al. SIOP‐PODC adapted risk stratification and treatment guidelines: recommendations for neuroblastoma in low‐ and middle‐income settings. Pediatr Blood Cancer. 2015;62:1305‐1316. 25810263 10.1002/pbc.25501 PMC5132052

[26] Parkes J , Hendricks M , Ssenyonga P , et al. SIOP PODC adapted treatment recommendations for standard‐risk medulloblastoma in low and middle income settings. Pediatr Blood Cancer. 2015;62:553‐564. 25418957 10.1002/pbc.25313

[27] Anderson BO . Evidence‐based methods to address disparities in global cancer control: the development of guidelines in Asia. Lancet Oncol. 2013;14:1154‐1155. 24176556 10.1016/S1470-2045(13)70496-0

[28] Shastri SS , Temin S , Almonte M , et al. Secondary prevention of cervical cancer: ASCO resource‐stratified guideline update. JCO Glob Oncol. 2022;8:e2200217. 36162041 10.1200/GO.22.00217 PMC9812449

[29] Chuang LT , Temin S , Berek JS , for the Management and Care of Patients with Invasive Cervical Cancer Resource‐Stratified Guideline Expert Panel . Management and care of patients with invasive cervical cancer: ASCO resource‐stratified guideline rapid recommendation update. JCO Glob Oncol. 2022;8:e2200027. 35245079 10.1200/GO.22.00027 PMC8920468

[30] Vanderpuye VD , Clemenceau JRV , Temin S , et al. Assessment of adult women with ovarian masses and treatment of epithelial ovarian cancer: ASCO resource‐stratified guideline. JCO Glob Oncol. 2021;7:1032‐1066. 34185571 10.1200/GO.21.00085 PMC8457806

[31] Chiorean EG , Nandakumar G , Fadelu T , et al. Treatment of patients with late‐stage colorectal cancer: ASCO resource‐stratified guideline. JCO Glob Oncol. 2020;6:414‐438. 32150483 10.1200/JGO.19.00367 PMC7124947

[32] Costas‐Chavarri A , Nandakumar G , Temin S , et al. Treatment of patients with early‐stage colorectal cancer: ASCO resource‐stratified guideline. J Glob Oncol. 2019;5:1‐19. 10.1200/JGO.18.00214 PMC6426503 30802158

[33] Lopes G , Stern MC , Temin S , et al. Early detection for colorectal cancer: ASCO resource‐stratified guideline. J Glob Oncol. 2019;5:1‐22. 10.1200/JGO.18.00213 PMC6426543 30802159

[34] Osman H , Shrestha S , Temin S , et al. Palliative care in the global setting: ASCO resource‐stratified practice guideline. J Glob Oncol. 2018;4:1‐24. 10.1200/JGO.18.00026 PMC6223509 30085844

[35] Jeronimo J , Castle PE , Temin S , et al. Secondary prevention of cervical cancer: ASCO resource‐stratified clinical practice guideline. J Glob Oncol. 2017;3:635‐657. 29094101 10.1200/JGO.2016.006577 PMC5646891

[36] Arrossi S , Temin S , Garland S , et al. Primary prevention of cervical cancer: American Society of Clinical Oncology resource‐stratified guideline. J Glob Oncol. 2017;3:611‐634. 29094100 10.1200/JGO.2016.008151 PMC5646902

[37] Munn Z , Peters MDJ , Stern C , Tufanaru C , McArthur A , Aromataris E . Systematic review or scoping review? Guidance for authors when choosing between a systematic or scoping review approach. BMC Med Res Methodol. 2018;18:143. 30453902 10.1186/s12874-018-0611-x PMC6245623

[38] Colquhoun HL , Levac D , O'Brien KK , et al. Scoping reviews: time for clarity in definition, methods, and reporting. J Clin Epidemiol. 2014;67:1291‐1294. 25034198 10.1016/j.jclinepi.2014.03.013

[39] Levac D , Colquhoun H , O'Brien KK . Scoping studies: advancing the methodology. Implement Sci. 2010;5:69. 20854677 10.1186/1748-5908-5-69 PMC2954944

[40] Tricco AC , Lillie E , Zarin W , et al. PRISMA extension for scoping reviews (PRISMA‐ScR): checklist and explanation. Ann Intern Med. 2018;169:467‐473. 30178033 10.7326/M18-0850

[41] National Institute for Health Research . PROSPERO. Accessed November 9, 2022. https://www.crd.york.ac.uk/prospero/

[42] Covidence . Accessed November 9, 2022. https://www.covidence.org

[43] World Bank . World Bank country and lending groups. Accessed November 9, 2022. https://datahelpdesk.worldbank.org/knowledgebase/articles/906519‐world‐bank‐country‐and‐lending‐groups

[44] Pankaj S , Nazneen S , Kumari A , et al. Myths and taboos—a major hindrance to cancer controls. “Inherited knowledge” a blessing or curse. Surgery after 21 cycles of chemotherapy “a Surgeon's ordeal”. Indian J Gynecol Oncol. 2018;16:1‐3.

[45] Abdullah A , Abdullah KL , Yip CH , Teo SH , Taib NA , Ng CJ . The decision‐making journey of Malaysian women with early breast cancer: a qualitative study. Asian Pac J Cancer Prev. 2013;14:7143‐7147. 24460266 10.7314/apjcp.2013.14.12.7143

[46] Sun H‐C , Zhu X‐D , Xu L , et al. Factors influencing adjuvant treatment decision making among Chinese patients with hepatocellular carcinoma (HCC): results of a patient survey. J Clin Oncol. 2021;39:346.33434057

[47] Wu X , Jiang Y‐N , Zhang Y‐L , et al. Impact of hematologists' personality and behavioral traits on medical decision‐making for elderly acute myeloid leukemia: a National Study in China. Blood. 2020;136:19.

[48] Zhou N , Liu F , Hu M , et al. Patient preference study on treatments of non small cell lung cancer in Western China. Value Health. 2018;21:S9.

[49] Lee PL , Cheong AT , Ng CJ , et al. Supporting patients in making treatment decisions for early prostate cancer—a qualitative study of healthcare professionals' views on barriers and challenges in an Asian country. J Men's Health. 2016;12:18‐24.

[50] Kowalski LP , Sanabria A . Priority setting in head and neck oncology in low‐resource environments. Curr Opin Otolaryngol Head Neck Surg. 2019;27:198‐202. 30870186 10.1097/MOO.0000000000000530

[51] Deliana M , Suza DE , Tarigan R . Advanced stage cancer patients experience in seeking treatment in Medan, Indonesia. Open Access Maced J Med Sci. 2019;7:2194‐2203. 31456851 10.3889/oamjms.2019.590 PMC6698112

[52] Brucker ME . Cultural considerations in palliative care‐oncology healthcare decision making‐ an observational study of familial influence in India and other cultures. Cancer Nurs. 2015;38:S52‐S53.

[53] Zhang L , Jiang M , Zhou Y , et al. Survey on breast cancer patients in China toward breast‐conserving surgery. Psychooncology. 2012;21:488‐495. 21322089 10.1002/pon.1922

[54] Doval DC , Kumar P , Talwar V , et al. Shared decision‐making and Medicolegal aspects: delivering high‐quality cancer Care in India. Indian J Palliat Care. 2020;26:405‐410. 33623298 10.4103/IJPC.IJPC_237_19 PMC7888410

[55] Demir Kureci H , Tanriverdi O , Ozcan M . Attitudes towards and experiences of ethical dilemmas in treatment decision‐making process among medical oncologists. J Eval Clin Pract. 2020;26:209‐215. 30912249 10.1111/jep.13127

[56] Agrawal S , Goel AK , Lal P . Participation in decision making regarding type of surgery and treatment‐related satisfaction in north Indian women with early breast cancer. J Cancer Res Ther. 2012;8:222‐225. 22842365 10.4103/0973-1482.98974

[57] Batistella GNR , Santos AJ , Paiva Neto MA , et al. Approaching glioblastoma during COVID‐19 pandemic: current recommendations and considerations in Brazil. Arq Neuropsiquiatr. 2021;79:167‐172. 33759984 10.1590/0004-282X-anp-2020-0434

[58] Wu X , Jiang YN , Zhang YL , et al. Impact of physicians' personalities and behavioral traits on treatment‐related decision‐making for elderly acute myeloid leukemia. J Gen Intern Med. 2021;36:3023‐3030. 33511569 10.1007/s11606-020-06467-w PMC8481415

[59] Liu JJ , Zhang S , Hao X , et al. Breast‐conserving therapy versus modified radical mastectomy: socioeconomic status determines who receives what—results from case‐control study in Tianjin, China. Cancer Epidemiol. 2012;36:89‐93. 21613000 10.1016/j.canep.2011.04.005

[60] Lin YP , Chen SZ , Yin WJ , et al. Factors that influencing patients' decision making for breast conserving surgery. Fudan Univ J Med Sci. 2008;25:641‐645.

[61] Gumus M , Ustaalioglu BO , Garip M , et al. Factors that affect Patients' decision‐making about mastectomy or breast conserving surgery, and the psychological effect of this choice on breast cancer patients. Breast Care (Basel). 2010;5:164‐168. 21048831 10.1159/000314266 PMC2931055

[62] Mishra A , Agarwal R , Tewari S , et al. Factors in decision making of breast conservation in early breast cancer: a study in northern India. Eur J Cancer. 2012;48:S209.

[63] Ogunkorode A , Holtslander L , Ferguson L , et al. Factors influencing the health‐seeking behaviors of women with advanced stages of breast cancer in southwestern Nigeria: an interpretive description study. Int J Africa Nurs Sci. 2021;14:100273.

[64] Obeidat RF , Masri MA , Marzouq M . Factors affecting Jordanian Women's surgical treatment decisions for early‐stage breast cancer. Asia Pac J Oncol Nurs. 2021;8:711‐719. 34790855 10.4103/apjon.apjon-20105 PMC8522584

[65] Rosabal‐Obando M , Osorio DS , Lassaletta A , et al. Follow‐up evaluation of a web‐based pediatric brain tumor board in Latin America. Pediatr Blood Cancer. 2021;68:e29073. 34003601 10.1002/pbc.29073

[66] Mailankody S , Kumar VS , Khan SA , Banavali SD , Bajpai J . Resource‐appropriate selection of osteosarcoma treatment protocols in low‐ and middle‐income countries. Pediatr Blood Cancer. 2022;69:e29540. 34971016 10.1002/pbc.29540

[67] Behan JM , Arora RS , Carnevale FA , Bakhshi S , Bhattacharjee B , Tsimicalis A . An ethnographic study of the moral experiences of children with cancer in New Delhi, India. Glob Qual Nurs Res. 2021;8:1‐14. 10.1177/2333393621995814 PMC7905724 33748333

[68] Bhandari D , Ozaki A , Ghimire B , et al. Oncology clinical practice guidelines usage among physicians in Nepal. J Eval Clin Pract. 2022;28:142‐150. 34184374 10.1111/jep.13594

[69] Yuksel E , Guven HE , Dogan L . Patients' perspective: what has changed in deciding about breast‐conserving surgery for early‐stage breast cancer in Turkey? Oncol Res Treat. 2018;41:744‐749. 30419566 10.1159/000492586

[70] De Guzman BG , Cabaya NF , Ting FIL , et al. Factors influencing treatment decisions among breast cancer patients in the Philippine general hospital cancer institute: medical oncology outpatient clinic. Ann Oncol. 2019;30:ix120.

[71] Teh YC , Shaari NE , Taib NA , et al. Determinants of choice of surgery in Asian patients with early breast cancer in a middle income country. Asian Pac J Cancer Prev. 2014;15:3163‐3167. 24815464 10.7314/apjcp.2014.15.7.3163

[72] Abbasi AN , Qureshi BM , Karim MU . Impact of multidisciplinary team meetings and decision‐making on cancer management in lower and middle income countries. Chest. 2021;159:887‐888. 10.1016/j.chest.2020.08.2097 33563448

[73] Shariff Z , Mansor AZ , Muhamad M . Decision making in breast cancer treatment—a qualitative inquiry. Pertanika J Soc Sci Humanit. 2008;16:269‐278.

[74] Harris JJ , Shao J , Sugarman J . Disclosure of cancer diagnosis and prognosis in northern Tanzania. Soc Sci Med. 2003;56:905‐913. 12593865 10.1016/s0277-9536(02)00090-4

[75] Skelton M , Alameddine R , Saifi O , et al. High‐cost cancer treatment across borders in conflict zones: experience of Iraqi patients in Lebanon. JCO Glob Oncol. 2020;6:59‐66. 32031440 10.1200/JGO.19.00281 PMC6998032

[76] Tang L . Barriers to effective decision making in cancer patient. Asia‐Pac J Clin Oncol. 2012;8:297.

[77] Pereira da Veiga CR , Pereira da Veiga C , Drummond‐Lage AP , Alves Wainstein AJ , Cristina de Melo A . Journey of the patient with melanoma: understanding resource use and bridging the gap between dermatologist, surgeon, and oncologist in different health care systems. J Glob Oncol. 2019;5:1‐8. 10.1200/JGO.19.00022 PMC6690632 31283414

[78] Amaro CP , Gomes L , Almeida D , Reiner K , Almeida GFG , Cruz MRS . Impact of multidisciplinary discussion on therapeutic decision in cancer patients: a prospective observational study. J Clin Oncol. 2017;35:e18243.

[79] Al‐Tabba A , Al‐Hussaini M , Mansour R , et al. Ethical considerations for treating cancer patients during the SARS‐CoV‐2 virus crisis: to treat or not to treat? A literature review and perspective from a cancer center in low‐middle income country. Front Med (Lausanne). 2020;7:561168. 33163499 10.3389/fmed.2020.561168 PMC7580805

[80] Bhatla N , Singhal S . The COVID‐19 pandemic and implications for gynaecologic cancer care. Indian J Gynecol Oncol. 2020;18:48. 32974417 10.1007/s40944-020-00395-7 PMC7180676

[81] Kilicarslan‐Toruner E , Akgun‐Citak E . Information‐seeking behaviours and decision‐making process of parents of children with cancer. Eur J Oncol Nurs. 2013;17:176‐183. 22521446 10.1016/j.ejon.2012.03.001

[82] Aziato L , Clegg‐Lamptey JN . Breast cancer diagnosis and factors influencing treatment decisions in Ghana. Health Care Women Int. 2015;36:543‐557. 24750095 10.1080/07399332.2014.911299

[83] Muhamad M , Afshari M , Kazilan F . Family support in cancer survivorship. Asian Pacific J Cancer Prev. 2011;12:1389‐1397. 22126470

[84] Kouya F , Picton S , Squire R , et al. Establishing a virtual multidisciplinary team meeting between Cameroon, Central Africa, and Leeds, UK, childhood cancer services. Pediatr Blood Cancer. 2021;S68‐S69.

[85] Wang T , Molassiotis A , Chung BPM , Zheng SL , Huang HQ , Tan JY . A qualitative exploration of the unmet information needs of Chinese advanced cancer patients and their informal caregivers. BMC Palliat Care. 2021;20:83. 34098905 10.1186/s12904-021-00774-7 PMC8186148

[86] Salisu WJ , Mirlashari J , Seylani K , Varaei S , Thorne S . Fatalism, distrust, and breast cancer treatment refusal in Ghana. Can Oncol Nurs J. 2022;32:198‐205. 35582248 10.5737/23688076322198205 PMC9040785

[87] Agyemang LS , Foster C , McLean C , Fenlon D , Wagland R . The cultural and structural influences that 'hide' information from women diagnosed with breast cancer in Ghana: an ethnography. BMC Womens Health. 2021;21:364. 34654413 10.1186/s12905-021-01502-2 PMC8518148

[88] Ahmadnia S , Ghalibaf AK , Kamkar S , Mohamadzadeh Z , Ghalibafian M . Survivor and parent engagement in childhood cancer treatment in Iran. Ecancermedicalscience. 2021;15:1220. 34158824 10.3332/ecancer.2021.1220 PMC8183647

[89] Gielen J , Bhatnagar S , Mishra S , et al. Can curative or life‐sustaining treatment be withheld or withdrawn? The opinions and views of Indian palliative‐care nurses and physicians. Med Health Care Philos. 2011;14:5‐18. 20676775 10.1007/s11019-010-9273-0

[90] El‐Hadidy MA , Elnahas W , Hegazy MA , et al. Psychiatric morbidity among Egyptian breast cancer patients and their partners and its impact on surgical decision‐making. Breast Cancer (Dove Med Press). 2012;4:25‐32. 24367191 10.2147/BCTT.S29890 PMC3846715

[91] Gong N , Du Q , Lou H , et al. Treatment decision‐making for older adults with cancer: a qualitative study. Nurs Ethics. 2021;28:242‐252. 32909913 10.1177/0969733020945752

[92] Ramakrishnan V , Kirushnakumar KS , Rathinam K , et al. Factors affecting treatment options in patients with advanced cancer of oral cavity—a single institution prospective study from southern India. Asia‐Pac J Clin Oncol. 2012;8:294‐295.

[93] Salek M , Force LM , Hlatwayo L , et al. An approach to understanding clinician treatment decision‐making at diagnosis in pediatric cancer: exploring challenges faced by clinicians in Zimbabwe. Pediatr Blood Cancer. 2021;68:S346.

[94] Hurdle V , Ouellet JF , Dixon E , et al. Does regional variation impact decision‐making in the management and palliation of pancreatic head adenocarcinoma? Results from an international survey. Can J Surg. 2014;57:E69‐E74. 24869619 10.1503/cjs.011213 PMC4035408

[95] Baijal G , Gupta T , Hotwani C , et al. Impact of comorbidity on therapeutic decision‐making in head and neck cancer: audit from a comprehensive cancer center in India. Head Neck. 2012;34:1251‐1254. 22076917 10.1002/hed.21897

[96] Menon MP , Coghill A , Mutyaba I , et al. Treatment recommendations for patients with NHL at the Uganda cancer institute. Blood. 2013;122:2960.

[97] Pawar D , Ahmed R , Chaudhari S . A survey of on platinum ineligible in head and neck cancer patients in India. Ann Oncol. 2017;28:x161.

[98] Jain A , Singh C , Dhawan R , et al. How to use a prioritised approach for treating hematological disorders during the COVID‐19 pandemic in India? Indian J Hematol Blood Transfus. 2020;36:605‐615. 32837051 10.1007/s12288-020-01300-0 PMC7274942

[99] Peruzzo N , Coelho JE , Gossling G , et al. Treatment delay and outcomes in stage IV lung cancer: the reality of a public hospital in a developing country. J Clin Oncol. 2019;37:e20709.

[100] Ngorsuraches S , Thongkeaw K . Patients' preferences and willingness‐to‐pay for postmenopausal hormone receptor‐positive, HER2‐negative advanced breast cancer treatments after failure of standard treatments. Springerplus. 2015;4:674. 26558177 10.1186/s40064-015-1482-9 PMC4635317

[101] Zhou N , Liu F , Jing W , Zhou J , Hu M . Study on Physicians' preference for treatment of NSCLC in Western China. Value Health. 2019;22:S111‐S112.

[102] Ramesh A , Ssoundarajan R . Cancer care during the COVID‐19 pandemic in southern India. Clin Cancer Res. 2020;26:PO‐008.

[103] Malhotra P , Yanamandra U , Kumar S , das S , Varma S . Common reasons for change of Chemoregimens in multiple myeloma: real world, comparison of two tertiary care centers. Clinl Lymphoma Myeloma Leuk. 2019;19:e223.

[104] Del Pilar E‐DM , Bonadio RC , Miranda VC , et al. Management of cervical cancer patients during the COVID‐19 pandemic: a challenge for developing countries. Ecancermedicalscience. 2020;14:1060. 32582375 10.3332/ecancer.2020.1060 PMC7302891

[105] Yousef MH , Alhalaseh YN , Mansour R , et al. The fair allocation of scarce medical resources: a comparative study from Jordan. Front Med (Lausanne). 2020;7:603406. 33585506 10.3389/fmed.2020.603406 PMC7873904

[106] Hong D , Zhou C , He H , Wang Y , Lu J , Hu S . A 10‐year follow‐up survey of treatment abandonment of children with acute myeloid leukemia in Suzhou, China. J Pediatr Hematol Oncol. 2016;38:437‐442. 27322718 10.1097/MPH.0000000000000601

[107] Mendoza MJL , Tan HNC , Hernandez ARB , et al. Medical oncology care amidst the COVID‐19 pandemic at the National University Hospital in The Philippines. Ecancermedicalscience. 2020;14:1066. 32728382 10.3332/ecancer.2020.1066 PMC7373646

[108] Siavashpour Z , Taghizadeh‐Hesary F , Rakhsha A . Recommendations on management of locally advanced rectal cancer during the COVID‐19 pandemic: an Iranian consensus. J Gastrointest Cancer. 2020;51:800‐804. 32656628 10.1007/s12029-020-00454-4 PMC7355082

[109] Vanderpuye V , Elhassan MMA , Simonds H . Preparedness for COVID‐19 in the oncology community in Africa. Lancet Oncol. 2020;21:621‐622. 32251623 10.1016/S1470-2045(20)30220-5 PMC7270461

[110] Pritchard‐Jones K , Abib S , Esiashvili N , et al. The threat of the COVID‐19 pandemic on reversing global life‐saving gains in the survival of childhood cancer: a call for collaborative action from SIOP, IPSO, PROS, WCC, CCI, St Jude global, UICC and WHPCA. Ecancer. 2021;15:1187. 10.3332/ecancer.2021.1187 PMC7987488 33777180

[111] Nair CK , Avaronnan M , Shenoy PK , et al. Impact of active tuberculosis on treatment decisions in cancer. Curr Probl Cancer. 2021;45:100643. 32972770 10.1016/j.currproblcancer.2020.100643

[112] Agom DA , Allen S , Neill S , et al. Social and health system complexities impacting on decision‐making for utilization of oncology and palliative Care in an African Context: a qualitative study. J Palliat Care. 2020;35:185‐191. 31842664 10.1177/0825859719892084

[113] Shelal Z , Alawad AS , Sun C , et al. Practicing oncology amidst war and religious strife‐how do physician beliefs affect care and work‐related stress? Support Care Cancer. 2012;20(Suppl 1):S196.

[114] Daroudi R , Mirzania M , Zendehdel K . Attitude of Iranian medical oncologists toward economic aspects, and policy‐making in relation to new cancer drugs. Int J Health Policy Manag. 2015;5:99‐105. 26927395 10.15171/ijhpm.2015.186 PMC4737548

[115] Li X , Dong M , Wen J‐y , et al. Cancer patients' awareness and role in family‐based medical decision‐making mode in Confucian area. J Clin Oncol. 2014;32:227.

[116] Alexander A , Kaluve R , Prabhu JS , et al. The impact of breast cancer on the patient and the family in Indian perspective. Indian J Palliat Care. 2019;25:66‐72. 30820105 10.4103/IJPC.IJPC_158_18 PMC6388591

[117] Bhattacharya M , Hamilton EP , Zafar Y . Oncologists' perceptions of cost and cancer care in India: a comparison of private practice (PPOs) and non‐private practice oncologists (NPPOs). J Clin Oncol. 2013;31:e17562.

[118] Soltani L , Khoshnood Z . Social support needs in patients with cancer. Middle East J Cancer. 2021;12:429‐438.

[119] Datta SS , Tripathi L , Varghese R , et al. Pivotal role of families in doctor‐patient communication in oncology: a qualitative study of patients, their relatives and cancer clinicians. Eur J Cancer Care (Engl). 2017;26:e12543. 10.1111/ecc.12543 27430633

[120] Alexander A , Kaluve R , Prabhu JS , et al. Treatment decision making, and strategies for coping with financial stress in Indian women diagnosed with breast cancer and their families. Cancer Res. 2018;78:P4‐10‐12.

[121] Olasehinde O , Arije O , Wuraola FO , et al. Life without a breast: exploring the experiences of young Nigerian women after mastectomy for breast cancer. J Glob Oncol. 2019;5:1‐6. 10.1200/JGO.18.00248 PMC6550027 31095453

[122] Brown O , Goliath V , van Rooyen DRM , Aldous C , Marais LC . Cultural factors that influence the treatment of osteosarcoma in Zulu patients: healthcare professionals' perspectives and strategies. Health SA. 2018;23:1095. 31934385 10.4102/hsag.v23i0.1095 PMC6917416

[123] Pramesh CS , Badwe RA , Bhoo‐Pathy N , et al. Priorities for cancer research in low‐ and middle‐income countries: a global perspective. Nat Med. 2022;28:649‐657. 35440716 10.1038/s41591-022-01738-x PMC9108683

[124] Iseli T , Fischer GF , Panje CM , et al. Insular decision criteria in clinical practice: analysis of decision‐making in oncology. Oncology. 2020;98:438‐444. 32428914 10.1159/000508132

