# Supplementary material for: Factors influencing treatment decision‐making for cancer patients in low‐ and middle‐income countries: A scoping review
Source: Cancer Med. 2023 Aug 1;12(17):18133–52. doi: 10.1002/cam4.6375 (PMC10524036; doi:10.1002/cam4.6375)
Supplement: Supplementary file 1 — Data S1. Supporting Information. [file CAM4-12-18133-s001.docx]

**Supplemental Tables for Manuscript: Factors Influencing Treatment Decision-Making for Cancer Patients in Low- and Middle-Income Countries: A Scoping Review, Salek et al.**

Supplemental Table 1. Search Strategy.

| **Database: CINAHL** | |
| --- | --- |
| 1. | (MH "Neoplasms") OR "neoplas*" OR "Tumor*" OR "Cancer*" OR "Malignan*" |
| 2. | (MH "Decision Making") OR (MH "Decision Making, Clinical") OR (MH "Decision Making, Ethical") OR (MH "Decision Making, Organizational") OR "decision making" OR "decision support" |
| 3. | (MH "Hormone Replacement Therapy") OR (MH "Chemotherapy, Cancer") OR (MH "Drug Delivery Systems") OR (MH "Drug Therapy, Combination") OR (MH "Hormone Therapy") OR (MH "Palliative Care") OR (MH "Bone Marrow Transplantation") OR (MH "Bone Marrow Transplantation, Allogeneic") OR (MH "Bone Marrow Transplantation, Autologous") OR "treatment" OR "chemotherap*" OR "cancer therap*" OR "palliative chemotherap*" OR "palliative radiation" OR "palliative surger*" OR "Palliative Treatment*" OR "Palliative Therap*" OR "palliative care" OR "hormone therap*" OR "drug maintenance" OR "maintenance treatment" OR "maintenance therap*" OR "bone marrow graft*" OR "bone marrow transplant*" OR "hormonal therap*" OR "hormone treatment*" |
| 4. | "developing country" OR "developing countries" OR "developing nation" OR "developing nations" OR "developing population" OR "developing populations" OR "developing world" OR "less developed country" OR "less developed countries" OR "less developed nation" OR "less developed nations" OR "less developed population" OR "less developed populations" OR "less developed world" OR "lesser developed country" OR "lesser developed countries" OR "lesser developed nation" OR "lesser developed nations" OR "lesser developed population" OR "lesser developed populations" OR "lesser developed world" OR "under developed country" OR "under developed countries" OR "under developed nation" OR "under developed nations" OR "under developed population" OR "under developed populations" OR "under developed world" OR "underdeveloped country" OR "underdeveloped countries" OR "underdeveloped nation" OR "underdeveloped nations" OR "underdeveloped population" OR "underdeveloped populations" OR "underdeveloped world" OR "middle income country" OR "middle income countries" OR "middle income nation" OR "middle income nations" OR "middle income population" OR "middle income populations" OR "low income country" OR "low income countries" OR "low income nation" OR "low income nations" OR "low income population" OR "low income populations" OR "lower income country" OR "lower income countries" OR "lower income nation" OR "lower income nations" OR "lower income population" OR "lower income populations" OR "underserved country" OR "underserved countries" OR "underserved nation" OR "underserved nations" OR "underserved population" OR "underserved populations" OR "underserved world" OR "under served country" OR "under served countries" OR "under served nation" OR "under served nations" OR "under served population" OR "under served populations" OR "under served world" OR "deprived country" OR "deprived countries" OR "deprived nation" OR "deprived nations" OR "deprived population" OR "deprived populations" OR "deprived world" OR "poor country" OR "poor countries" OR "poor nation" OR "poor nations" OR "poor population" OR "poor populations" OR "poor world" OR "poorer country" OR "poorer countries" OR "poorer nation" OR "poorer nations" OR "poorer population" OR "poorer populations" OR "poorer world" OR "developing economy" OR "developing economies" OR "less developed economy" OR "less developed economies" OR "lesser developed economy" OR "lesser developed economies" OR "under developed economy" OR "under developed economies" OR "underdeveloped economy" OR "underdeveloped economies" OR "middle income economy" OR "middle income economies" OR "low income economy" OR "low income economies" OR "lower income economy" OR "lower income economies" OR "low gdp" OR "low gnp" OR "low gross domestic" OR "low gross national" OR "lower gdp" OR "lower gnp" OR "lower gross domestic" OR "lower gross national" OR lmic OR lmics OR "third world" OR "lami country" OR "lami countries" OR "transitional country" OR "transitional countries" OR Africa OR Asia OR Caribbean OR "West Indies" OR "South America" OR "Latin America" OR "Central America" OR "atlantic islands" OR "commonwealth of independent states" OR "pacific islands" OR "indian ocean islands" OR "eastern europe" OR "Baltic States" OR Afghanistan OR Albania OR Algeria OR Angola OR Antigua OR Barbuda OR Argentina OR Armenia OR Armenian OR Aruba OR Azerbaijan OR Bahrain OR Bangladesh OR Barbados OR Benin OR Byelarus OR Byelorussian OR Belarus OR Belorussian OR Belorussia OR Belize OR Bhutan OR Bolivia OR Bosnia OR Herzegovina OR Hercegovina OR Botswana OR Brasil OR Brazil OR Bulgaria OR "Burkina Faso" OR "Burkina Fasso" OR "Upper Volta" OR Burundi OR Urundi OR Cambodia OR "Khmer Republic" OR Kampuchea OR Cameroon OR Cameroons OR Cameron OR Camerons OR "Cabo Verde" OR "Cape Verde" OR "Central African Republic" OR Chad OR Chile OR China OR Colombia OR Comoros OR "Comoro Islands" OR Comores OR Mayotte OR Congo OR Zaire OR "Costa Rica" OR "Cote d`Ivoire" OR "Ivory Coast" OR Croatia OR Cuba OR Cyprus OR Czechoslovakia OR "Czech Republic" OR Slovakia OR "Slovak Republic" OR Djibouti OR "French Somaliland" OR Dominica OR "Dominican Republic" OR "East Timor" OR "East Timur" OR "Timor Leste" OR Ecuador OR Egypt OR "United Arab Republic" OR "El Salvador" OR Eritrea OR Estonia OR Ethiopia OR Eswatini OR Fiji OR Gabon OR "Gabonese Republic" OR Gambia OR Gaza OR "Georgia Republic" OR "Georgian Republic" OR Ghana OR "Gold Coast" OR Greece OR Grenada OR Guatemala OR Guinea OR Guam OR Guiana OR Guyana OR Haiti OR Honduras OR Hungary OR India OR Maldives OR Indonesia OR Iran OR Iraq OR "Isle of Man" OR Jamaica OR Jordan OR Kazakhstan OR Kazakh OR Kenya OR Kiribati OR Nauru OR Korea OR Kosovo OR Kyrgyzstan OR Kirghizia OR "Kyrgyz Republic" OR Kirghiz OR Kirgizstan OR "Lao PDR" OR Laos OR Latvia OR Lebanon OR Lesotho OR Basutoland OR Liberia OR Libya OR Lithuania OR Macedonia OR Madagascar OR "Malagasy Republic" OR Malaysia OR Malaya OR Malay OR Sabah OR Sarawak OR Malawi OR Nyasaland OR Mali OR Malta OR "Marshall Islands" OR Mauritania OR Mauritius OR melanesia OR "Agalega Islands" OR Mexico OR Micronesia OR "Middle East" OR Moldova OR Moldovia OR Moldovian OR Mongolia OR Montenegro OR Morocco OR Ifni OR Mozambique OR Myanmar OR Myanma OR Burma OR Namibia OR Nepal OR "Netherlands Antilles" OR "New Caledonia" OR Nicaragua OR Niger OR Nigeria OR "Northern Mariana Islands" OR Oman OR Muscat OR Pakistan OR Palau OR Palestine OR Panama OR Paraguay OR Peru OR Philippines OR Philipines OR Phillipines OR Phillippines OR Poland OR Portugal OR 'Puerto Rico' OR Romania OR Rumania OR Roumania OR Russia OR Russian OR Rwanda OR Ruanda OR "Saint Kitts" OR "St Kitts" OR Nevis OR "Saint Lucia" OR "St Lucia" OR "Saint Vincent" OR "St Vincent" OR Grenadines OR Samoa OR "Samoan Islands" OR "Navigator Island" OR "Navigator Islands" OR "Sao Tome" OR "Saudi Arabia" OR Senegal OR Serbia OR Montenegro OR Seychelles OR "Sierra Leone" OR Slovenia OR "Sri Lanka" OR Ceylon OR "Solomon Islands" OR Somalia OR Sudan OR Suriname OR Surinam OR Swaziland OR Syria OR Syrian OR Tajikistan OR Tadzhikistan OR Tadjikistan OR Tadzhik OR Tanzania OR Thailand OR Togo OR "Togolese Republic" OR Tonga OR Trinidad OR Tobago OR Tunisia OR Turkey OR Turkmenistan OR Turkmen OR Tuvalu OR Uganda OR Ukraine OR Uruguay OR USSR OR "Soviet Union" OR "Union of Soviet Socialist Republics" OR Uzbekistan OR Uzbek OR Vanuatu OR "New Hebrides" OR Venezuela OR Vietnam OR "Viet Nam" OR "West Bank" OR Yemen OR Yugoslavia OR Zambia OR Zimbabwe OR Rhodesia OR (MH "Developing Countries") OR (MH "Africa") OR (MH "Africa, Northern") OR (MH "Africa South of the Sahara") OR (MH "Africa, Central") OR (MH "Africa, Eastern") OR (MH "Africa, Southern") OR (MH "Africa, Western") OR (MH "Asia") OR (MH "Asia, Central") OR (MH "Asia, Southeastern") OR (MH "Asia, Western") OR (MH "Far East") OR (MH "West Indies") OR (MH "South America") OR (MH "Latin America") OR (MH "Central America") OR (MH "Atlantic Islands") OR (MH "Commonwealth of Independent States") OR (MH "Pacific Islands") OR (MH "Indian Ocean Islands") OR (MH "Mediterranean Islands") OR (MH "Europe, Eastern") OR (MH "Afghanistan") OR (MH "Albania") OR (MH "Algeria") OR (MH "American Samoa") OR (MH "Angola") OR (MH "Antigua") OR (MH "Argentina") OR (MH "Armenia") OR (MH "Azerbaijan") OR (MH "Bahrain") OR (MH "Baltic States+") OR (MH "Bangladesh") OR (MH "Barbados") OR (MH "Belize") OR (MH "Benin") OR (MH "Bhutan") OR (MH "Bolivia") OR (MH "Bosnia-Herzegovina") OR (MH "Botswana") OR (MH "Brazil") OR (MH "Bulgaria") OR (MH "Burkina Faso") OR (MH "Burundi") OR (MH "Byelarus") OR (MH "Cambodia") OR (MH "Cameroon") OR (MH "Cape Verde") OR (MH "Central African Republic") OR (MH "Chad") OR (MH "Chile") OR (MH "China+") OR (MH "Colombia") OR (MH "Congo") OR (MH "Costa Rica") OR (MH "Cote d'Ivoire") OR (MH "Croatia") OR (MH "Cuba") OR (MH "Czech Republic") OR (MH "Czechoslovakia+") OR (MH "Democratic Republic of the Congo") OR (MH "Djibouti") OR (MH "Dominica") OR (MH "Dominican Republic") OR (MH "East Timor") OR (MH "Ecuador") OR (MH "Egypt") OR (MH "El Salvador") OR (MH "Equatorial Guinea") OR (MH "Eritrea") OR (MH "Estonia") OR (MH "Ethiopia") OR (MH "French Guiana") OR (MH "Gabon") OR (MH "Gambia") OR (MH "Georgia (Republic)") OR (MH "Ghana") OR (MH "Greece") OR (MH "Guam") OR (MH "Guatemala") OR (MH "Guinea") OR (MH "Guinea-Bissau") OR (MH "Guyana") OR (MH "Haiti") OR (MH "Honduras") OR (MH "Hungary") OR (MH "India") OR (MH "Indonesia") OR (MH "Iran") OR (MH "Iraq") OR (MH "Jamaica") OR (MH "Jordan") OR (MH "Kazakhstan") OR (MH "Kenya") OR (MH "Korea") OR (MH "Kyrgyzstan") OR (MH "Laos") OR (MH "Latvia") OR (MH "Lebanon") OR (MH "Lesotho") OR (MH "Liberia") OR (MH "Libya") OR (MH "Lithuania") OR (MH "Macedonia (Republic)") OR (MH "Madagascar") OR (MH "Malawi") OR (MH "Malaysia") OR (MH "Mali") OR (MH "Mauritania") OR (MH "Melanesia+") OR (MH "Mexico") OR (MH "Micronesia+") OR (MH "Middle East") OR (MH "Moldova") OR (MH "Mongolia") OR (MH "Morocco") OR (MH "Mozambique") OR (MH "Myanmar") OR (MH "Namibia") OR (MH "Nepal") OR (MH "Netherlands Antilles") OR (MH "Nicaragua") OR (MH "Niger") OR (MH "Nigeria") OR (MH "North Korea") OR (MH "Oman") OR (MH "Pakistan") OR (MH "Panama+") OR (MH "Papua New Guinea") OR (MH "Paraguay") OR (MH "Peru") OR (MH "Philippines") OR (MH "Poland") OR (MH "Polynesia") OR (MH "Portugal") OR (MH "Puerto Rico") OR (MH "Romania") OR (MH "Russia") OR (MH "Rwanda") OR (MH "Samoa+")OR (MH "Saudi Arabia") OR (MH "Senegal") OR (MH "Serbia") OR (MH "Sierra Leone") OR (MH "Slovakia") OR (MH "Slovenia") OR (MH "Somalia") OR (MH "South Africa") OR (MH "South Korea") OR (MH "Sri Lanka") OR (MH "Sudan") OR (MH "Suriname") OR (MH "Swaziland") OR (MH "Syria") OR (MH "Tajikistan") OR (MH "Tanzania") OR (MH "Thailand") OR (MH "Togo") OR (MH "Trinidad and Tobago") OR (MH "Tunisia") OR (MH "Turkey") OR (MH "Turkmenistan") OR (MH "Uganda") OR (MH "Ukraine") OR (MH "Uruguay") OR (MH "USSR+") OR (MH "Uzbekistan") OR (MH "Venezuela") OR (MH "Vietnam") OR (MH "Western Samoa") OR (MH "Yemen") OR (MH "Yugoslavia+") OR (MH "Zambia") OR (MH "Zimbabwe") |
| 5. | #1 AND #2 AND #3 AND #4 |
| **Database: Embase** | |
| **1.** | 'malignant neoplasm'/exp OR ('cancer*' OR 'malignant neoplas*' OR 'malignant neoplastic disease*' OR 'malignant tumo*' OR 'oncology'):ab,ti,kw |
| **2.** | 'decision making'/exp OR 'shared decision making'/exp OR ('decision making' OR 'decision process*' OR 'clinical decision support'):ab,ti,kw |
| **3.** | 'treatment'/exp OR 'cancer therapy'/exp OR 'cancer chemotherapy'/exp OR 'intervention'/exp OR 'palliative therapy'/exp OR 'maintenance therapy'/exp OR 'metronomic drug administration'/exp OR 'bone marrow transplantation'/exp OR 'hormonal therapy'/exp OR ('therap*' OR 'treatment*' OR 'chemotherap*' OR 'intervention*' OR 'cancer therap*' OR 'palliative care' OR 'palliative consultation' OR 'palliative medicine' OR 'palliative radiotherapy*' OR 'palliative surgery' OR 'palliative treatment*' OR 'drug maintenance' OR 'maintenance treatment*' OR 'maintenance therap*' OR 'metronomic administration' OR 'bone marrow graft*' OR 'bone marrow transplant*' OR 'hormone therap*' OR 'hormone treatment*'):ab,ti,kw |
| **4.** | 'developing country':ab,ti,kw OR 'developing countries':ab,ti,kw OR 'developing nation':ab,ti,kw OR 'developing nations':ab,ti,kw OR 'developing population':ab,ti,kw OR 'developing populations':ab,ti,kw OR 'developing world':ab,ti,kw OR 'less developed country':ab,ti,kw OR 'less developed countries':ab,ti,kw OR 'less developed nation':ab,ti,kw OR 'less developed nations':ab,ti,kw OR 'less developed population':ab,ti,kw OR 'less developed populations':ab,ti,kw OR 'less developed world':ab,ti,kw OR 'lesser developed country':ab,ti,kw OR 'lesser developed countries':ab,ti,kw OR 'lesser developed nation':ab,ti,kw OR 'lesser developed nations':ab,ti,kw OR 'lesser developed population':ab,ti,kw OR 'lesser developed populations':ab,ti,kw OR 'lesser developed world':ab,ti,kw OR 'under developed country':ab,ti,kw OR 'under developed countries':ab,ti,kw OR 'under developed nation':ab,ti,kw OR 'under developed nations':ab,ti,kw OR 'under developed population':ab,ti,kw OR 'under developed populations':ab,ti,kw OR 'under developed world':ab,ti,kw OR 'underdeveloped country':ab,ti,kw OR 'underdeveloped countries':ab,ti,kw OR 'underdeveloped nation':ab,ti,kw OR 'underdeveloped nations':ab,ti,kw OR 'underdeveloped population':ab,ti,kw OR 'underdeveloped populations':ab,ti,kw OR 'underdeveloped world':ab,ti,kw OR 'middle income country':ab,ti,kw OR 'middle income countries':ab,ti,kw OR 'middle income nation':ab,ti,kw OR 'middle income nations':ab,ti,kw OR 'middle income population':ab,ti,kw OR 'middle income populations':ab,ti,kw OR 'low income country':ab,ti,kw OR 'low income countries':ab,ti,kw OR 'low income nation':ab,ti,kw OR 'low income nations':ab,ti,kw OR 'low income population':ab,ti,kw OR 'low income populations':ab,ti,kw OR 'lower income country':ab,ti,kw OR 'lower income countries':ab,ti,kw OR 'lower income nation':ab,ti,kw OR 'lower income nations':ab,ti,kw OR 'lower income population':ab,ti,kw OR 'lower income populations':ab,ti,kw OR 'underserved country':ab,ti,kw OR 'underserved countries':ab,ti,kw OR 'underserved nation':ab,ti,kw OR 'underserved nations':ab,ti,kw OR 'underserved population':ab,ti,kw OR 'underserved populations':ab,ti,kw OR 'underserved world':ab,ti,kw OR 'under served country':ab,ti,kw OR 'under served countries':ab,ti,kw OR 'under served nation':ab,ti,kw OR 'under served nations':ab,ti,kw OR 'under served population':ab,ti,kw OR 'under served populations':ab,ti,kw OR 'under served world':ab,ti,kw OR 'deprived country':ab,ti,kw OR 'deprived countries':ab,ti,kw OR 'deprived nation':ab,ti,kw OR 'deprived nations':ab,ti,kw OR 'deprived population':ab,ti,kw OR 'deprived populations':ab,ti,kw OR 'deprived world':ab,ti,kw OR 'poor country':ab,ti,kw OR 'poor countries':ab,ti,kw OR 'poor nation':ab,ti,kw OR 'poor nations':ab,ti,kw OR 'poor population':ab,ti,kw OR 'poor populations':ab,ti,kw OR 'poor world':ab,ti,kw OR 'poorer country':ab,ti,kw OR 'poorer countries':ab,ti,kw OR 'poorer nation':ab,ti,kw OR 'poorer nations':ab,ti,kw OR 'poorer population':ab,ti,kw OR 'poorer populations':ab,ti,kw OR 'poorer world':ab,ti,kw OR 'developing economy':ab,ti,kw OR 'developing economies':ab,ti,kw OR 'less developed economy':ab,ti,kw OR 'less developed economies':ab,ti,kw OR 'lesser developed economy':ab,ti,kw OR 'lesser developed economies':ab,ti,kw OR 'under developed economy':ab,ti,kw OR 'under developed economies':ab,ti,kw OR 'underdeveloped economy':ab,ti,kw OR 'underdeveloped economies':ab,ti,kw OR 'middle income economy':ab,ti,kw OR 'middle income economies':ab,ti,kw OR 'low income economy':ab,ti,kw OR 'low income economies':ab,ti,kw OR 'lower income economy':ab,ti,kw OR 'lower income economies':ab,ti,kw OR 'low gdp':ab,ti,kw OR 'low gnp':ab,ti,kw OR 'low gross domestic':ab,ti,kw OR 'low gross national':ab,ti,kw OR 'lower gdp':ab,ti,kw OR 'lower gnp':ab,ti,kw OR 'lower gross domestic':ab,ti,kw OR 'lower gross national':ab,ti,kw OR lmic:ab,ti,kw OR lmics:ab,ti,kw OR 'third world':ab,ti,kw OR 'lami country':ab,ti,kw OR 'lami countries':ab,ti,kw OR 'transitional country':ab,ti,kw OR 'transitional countries':ab,ti,kw OR Africa:ab,ti,kw OR Asia:ab,ti,kw OR Caribbean:ab,ti,kw OR 'West Indies':ab,ti,kw OR 'South America':ab,ti,kw OR 'Latin America':ab,ti,kw OR 'Central America':ab,ti,kw OR 'atlantic islands':ab,ti,kw OR 'commonwealth of independent states':ab,ti,kw OR 'pacific islands':ab,ti,kw OR 'indian ocean islands':ab,ti,kw OR 'eastern europe':ab,ti,kw OR Afghanistan:ab,ti,kw OR Albania:ab,ti,kw OR Algeria:ab,ti,kw OR Angola:ab,ti,kw OR Antigua:ab,ti,kw OR Barbuda:ab,ti,kw OR Argentina:ab,ti,kw OR Armenia:ab,ti,kw OR Armenian:ab,ti,kw OR Aruba:ab,ti,kw OR Azerbaijan:ab,ti,kw OR Bahrain:ab,ti,kw OR Bangladesh:ab,ti,kw OR Barbados:ab,ti,kw OR Benin:ab,ti,kw OR Byelarus:ab,ti,kw OR Byelorussian:ab,ti,kw OR Belarus:ab,ti,kw OR Belorussian:ab,ti,kw OR Belorussia:ab,ti,kw OR Belize:ab,ti,kw OR Bhutan:ab,ti,kw OR Bolivia:ab,ti,kw OR Bosnia:ab,ti,kw OR Herzegovina:ab,ti,kw OR Hercegovina:ab,ti,kw OR Botswana:ab,ti,kw OR Brasil:ab,ti,kw OR Brazil:ab,ti,kw OR Bulgaria:ab,ti,kw OR 'Burkina Faso':ab,ti,kw OR 'Burkina Fasso':ab,ti,kw OR 'Upper Volta':ab,ti,kw OR Burundi:ab,ti,kw OR Urundi:ab,ti,kw OR Cambodia:ab,ti,kw OR 'Khmer Republic':ab,ti,kw OR Kampuchea:ab,ti,kw OR Cameroon:ab,ti,kw OR Cameroons:ab,ti,kw OR Cameron:ab,ti,kw OR Camerons:ab,ti,kw OR 'Cape Verde':ab,ti,kw OR 'Central African Republic':ab,ti,kw OR Chad:ab,ti,kw OR Chile:ab,ti,kw OR China:ab,ti,kw OR Colombia:ab,ti,kw OR Comoros:ab,ti,kw OR 'Comoro Islands':ab,ti,kw OR Comores:ab,ti,kw OR Mayotte:ab,ti,kw OR Congo:ab,ti,kw OR Zaire:ab,ti,kw OR 'Costa Rica':ab,ti,kw OR 'Cote d`Ivoire' OR 'Ivory Coast':ab,ti,kw OR Croatia:ab,ti,kw OR Cuba:ab,ti,kw OR Cyprus:ab,ti,kw OR Czechoslovakia:ab,ti,kw OR 'Czech Republic':ab,ti,kw OR Slovakia:ab,ti,kw OR 'Slovak Republic':ab,ti,kw OR Djibouti:ab,ti,kw OR 'French Somaliland':ab,ti,kw OR Dominica:ab,ti,kw OR 'Dominican Republic':ab,ti,kw OR 'East Timor':ab,ti,kw OR 'East Timur':ab,ti,kw OR 'Timor Leste':ab,ti,kw OR Ecuador:ab,ti,kw OR Egypt:ab,ti,kw OR 'United Arab Republic':ab,ti,kw OR El Salvador:ab,ti,kw OR Eritrea:ab,ti,kw OR Estonia:ab,ti,kw OR Ethiopia:ab,ti,kw OR Fiji:ab,ti,kw OR Gabon:ab,ti,kw OR 'Gabonese Republic':ab,ti,kw OR Gambia:ab,ti,kw OR Gaza:ab,ti,kw OR 'Georgia Republic':ab,ti,kw OR 'Georgian Republic':ab,ti,kw OR Ghana:ab,ti,kw OR 'Gold Coast':ab,ti,kw OR Greece:ab,ti,kw OR Grenada:ab,ti,kw OR Guatemala:ab,ti,kw OR Guinea:ab,ti,kw OR Guam:ab,ti,kw OR Guiana:ab,ti,kw OR Guyana:ab,ti,kw OR Haiti:ab,ti,kw OR Honduras:ab,ti,kw OR Hungary:ab,ti,kw OR India:ab,ti,kw OR Maldives:ab,ti,kw OR Indonesia:ab,ti,kw OR Iran:ab,ti,kw OR Iraq:ab,ti,kw OR 'Isle of Man':ab,ti,kw OR Jamaica:ab,ti,kw OR Jordan:ab,ti,kw OR Kazakhstan:ab,ti,kw OR Kazakh:ab,ti,kw OR Kenya:ab,ti,kw OR Kiribati:ab,ti,kw OR Korea:ab,ti,kw OR Kosovo:ab,ti,kw OR Kyrgyzstan:ab,ti,kw OR Kirghizia:ab,ti,kw OR 'Kyrgyz Republic':ab,ti,kw OR Kirghiz:ab,ti,kw OR Kirgizstan:ab,ti,kw OR 'Lao PDR':ab,ti,kw OR Laos:ab,ti,kw OR Latvia:ab,ti,kw OR Lebanon:ab,ti,kw OR Lesotho:ab,ti,kw OR Basutoland:ab,ti,kw OR Liberia:ab,ti,kw OR Libya:ab,ti,kw OR Lithuania:ab,ti,kw OR Macedonia:ab,ti,kw OR Madagascar:ab,ti,kw OR 'Malagasy Republic':ab,ti,kw OR Malaysia:ab,ti,kw OR Malaya:ab,ti,kw OR Malay:ab,ti,kw OR Sabah:ab,ti,kw OR Sarawak:ab,ti,kw OR Malawi:ab,ti,kw OR Nyasaland:ab,ti,kw OR Mali:ab,ti,kw OR Malta:ab,ti,kw OR 'Marshall Islands':ab,ti,kw OR Mauritania:ab,ti,kw OR Mauritius:ab,ti,kw OR melanesia:ab,ti,kw OR 'Agalega Islands':ab,ti,kw OR Mexico:ab,ti,kw OR Micronesia:ab,ti,kw OR 'Middle East':ab,ti,kw OR Moldova:ab,ti,kw OR Moldovia:ab,ti,kw OR Moldovian:ab,ti,kw OR Mongolia:ab,ti,kw OR Montenegro:ab,ti,kw OR Morocco:ab,ti,kw OR Ifni:ab,ti,kw OR Mozambique:ab,ti,kw OR Myanmar:ab,ti,kw OR Myanma:ab,ti,kw OR Burma:ab,ti,kw OR Namibia:ab,ti,kw OR Nepal:ab,ti,kw OR 'Netherlands Antilles':ab,ti,kw OR 'New Caledonia':ab,ti,kw OR Nicaragua:ab,ti,kw OR Niger:ab,ti,kw OR Nigeria:ab,ti,kw OR 'Northern Mariana Islands':ab,ti,kw OR Oman:ab,ti,kw OR Muscat:ab,ti,kw OR Pakistan:ab,ti,kw OR Palau:ab,ti,kw OR Palestine:ab,ti,kw OR Panama:ab,ti,kw OR Paraguay:ab,ti,kw OR Peru:ab,ti,kw OR Philippines:ab,ti,kw OR Philipines:ab,ti,kw OR Phillipines:ab,ti,kw OR Phillippines:ab,ti,kw OR Poland:ab,ti,kw OR Portugal:ab,ti,kw OR 'Puerto Rico':ab,ti,kw OR Romania:ab,ti,kw OR Rumania:ab,ti,kw OR Roumania:ab,ti,kw OR Russia:ab,ti,kw OR Russian:ab,ti,kw OR Rwanda:ab,ti,kw OR Ruanda:ab,ti,kw OR 'Saint Kitts':ab,ti,kw OR 'St Kitts':ab,ti,kw OR Nevis:ab,ti,kw OR 'Saint Lucia':ab,ti,kw OR 'St Lucia':ab,ti,kw OR 'Saint Vincent':ab,ti,kw OR 'St Vincent':ab,ti,kw OR Grenadines:ab,ti,kw OR Samoa:ab,ti,kw OR 'Samoan Islands':ab,ti,kw OR 'Navigator Island':ab,ti,kw OR 'Navigator Islands':ab,ti,kw OR 'Sao Tome':ab,ti,kw OR 'Saudi Arabia':ab,ti,kw OR Senegal:ab,ti,kw OR Serbia:ab,ti,kw OR Montenegro:ab,ti,kw OR Seychelles:ab,ti,kw OR 'Sierra Leone':ab,ti,kw OR Slovenia:ab,ti,kw OR 'Sri Lanka':ab,ti,kw OR Ceylon:ab,ti,kw OR 'Solomon Islands':ab,ti,kw OR Somalia:ab,ti,kw OR Sudan:ab,ti,kw OR Suriname:ab,ti,kw OR Surinam:ab,ti,kw OR Swaziland:ab,ti,kw OR Syria:ab,ti,kw OR Syrian:ab,ti,kw OR Tajikistan:ab,ti,kw OR Tadzhikistan:ab,ti,kw OR Tadjikistan:ab,ti,kw OR Tadzhik:ab,ti,kw OR Tanzania:ab,ti,kw OR Thailand:ab,ti,kw OR Togo:ab,ti,kw OR 'Togolese Republic':ab,ti,kw OR Tonga:ab,ti,kw OR Trinidad:ab,ti,kw OR Tobago:ab,ti,kw OR Tunisia:ab,ti,kw OR Turkey:ab,ti,kw OR Turkmenistan:ab,ti,kw OR Turkmen:ab,ti,kw OR Tuvalu:ab,ti,kw OR Uganda:ab,ti,kw OR Ukraine:ab,ti,kw OR Uruguay:ab,ti,kw OR USSR:ab,ti,kw OR 'Soviet Union':ab,ti,kw OR 'Union of Soviet Socialist Republics':ab,ti,kw OR Uzbekistan:ab,ti,kw OR Vanuatu:ab,ti,kw OR 'New Hebrides':ab,ti,kw OR Venezuela:ab,ti,kw OR Vietnam:ab,ti,kw OR 'Viet Nam':ab,ti,kw OR 'West Bank':ab,ti,kw OR Yemen:ab,ti,kw OR Yugoslavia:ab,ti,kw OR Zambia:ab,ti,kw OR Zimbabwe:ab,ti,kw OR Rhodesia:ab,ti,kw OR 'developing country'/exp OR 'Africa'/de OR 'Africa south of the Sahara'/de OR 'North Africa'/de OR 'Central Africa'/de OR 'Asia'/de OR 'South Asia'/de OR 'Southeast Asia'/de OR 'South America'/de OR 'Central America'/de OR 'South and Central America'/de OR 'Atlantic islands'/de OR 'Caribbean Islands'/de OR 'Pacific islands'/de OR 'Indian Ocean'/de OR 'Eastern Europe'/de OR Afghanistan/exp OR Albania/exp OR Algeria/exp OR 'American Samoa'/exp OR Angola/exp OR 'Antigua and Barbuda'/exp OR Argentina/exp OR Armenia/exp OR Azerbaijan/exp OR Bahrain/exp OR Bangladesh/exp OR Barbados/exp OR Benin/exp OR 'Belarus'/exp OR 'Baltic States'/exp OR Belize/exp OR Bhutan/exp OR Bolivia/exp OR 'Bosnia and Herzegovina'/exp OR Botswana/exp OR Brazil/exp OR Bulgaria/exp OR 'Burkina Faso'/exp OR Burundi/exp OR Cambodia/exp OR Cameroon/exp OR 'Cape Verde'/exp OR 'Central African Republic'/exp OR Chad/exp OR Chile/exp OR China/exp OR Colombia/exp OR Comoros/exp OR Congo/exp OR 'Costa Rica'/exp OR 'Cote d`Ivoire'/exp OR Croatia/exp OR Cuba/exp OR Cyprus/exp OR Czechoslovakia/exp OR 'Czech Republic'/exp OR Slovakia/exp OR Djibouti/exp OR 'Democratic Republic Congo'/exp OR Dominica/exp OR 'Dominican Republic'/exp OR 'Timor-Leste'/exp OR Ecuador/exp OR Egypt/exp OR 'El Salvador'/exp OR Eritrea/exp OR Estonia/exp OR 'Eswatini'/exp OR Ethiopia/exp OR 'French Guiana'/exp OR Fiji/exp OR Gabon/exp OR Gambia/exp OR 'Georgia (Republic) '/exp OR Ghana/exp OR Greece/exp OR Grenada/exp OR Guatemala/exp OR Guinea/exp OR Guinea-Bissau/exp OR Guam/exp OR Guyana/exp OR Haiti/exp OR Honduras/exp OR Hungary/exp OR India/exp OR Indonesia/exp OR Iran/exp OR Iraq/exp OR Jamaica/exp OR Jordan/exp OR Kazakhstan/exp OR Kenya/exp OR Korea/exp OR Kyrgyzstan/exp OR Laos/exp OR Latvia/exp OR Lebanon/exp OR Lesotho/exp OR Liberia/exp OR 'Libyan Arab Jamahiriya'/exp OR Lithuania/exp OR 'Macedonia (republic)'/exp OR Madagascar/exp OR Malaysia/exp OR Malawi/exp OR Mali/exp OR Malta/exp OR Mauritania/exp OR Mauritius/exp OR "Melanesia"/exp OR Mexico/exp OR 'Federated States of Micronesia'/exp OR 'Middle East'/de OR Moldova/exp OR Mongolia/exp OR Montenegro/exp OR Morocco/exp OR Mozambique/exp OR Myanmar/exp OR Namibia/exp OR Nepal/exp OR 'Netherlands Antilles'/exp OR 'New Caledonia'/exp OR Nicaragua/exp OR Niger/exp OR Nigeria/exp OR 'North Korea'/exp OR Oman/exp OR Pakistan/exp OR Palau/exp OR Panama/exp OR 'Papua New Guinea'/exp OR Paraguay/exp OR Peru/exp OR Philippines/exp OR Poland/exp OR Portugal/exp OR 'Puerto Rico'/exp OR Romania/exp OR 'Russian Federation'/exp OR Rwanda/exp OR 'Saint Kitts and Nevis'/exp OR 'Saint Lucia'/exp OR 'Saint Vincent and the Grenadines'/exp OR 'Samoan Islands'/exp OR Samoa/exp OR 'Saudi Arabia'/exp OR Senegal/exp OR Serbia/exp OR 'Montenegro (republic)'/exp OR Seychelles/exp OR 'Sierra Leone'/exp OR Slovenia/exp OR 'Sri Lanka'/exp OR Somalia/exp OR 'South Korea'/exp OR 'South Africa'/exp OR Sudan/exp OR Suriname/exp OR 'Syrian Arab Republic'/exp OR Tajikistan/exp OR Tanzania/exp OR Thailand/exp OR Togo/exp OR Tonga/exp OR 'Trinidad and Tobago'/exp OR Tunisia/exp OR 'Turkey (republic)'/exp OR Turkmenistan/exp OR Uganda/exp OR Ukraine/exp OR Uruguay/exp OR USSR/exp OR Uzbekistan/exp OR Vanuatu/exp OR Venezuela/exp OR 'Viet Nam'/exp OR Yemen/exp OR Yugoslavia/exp OR 'Yugoslavia (pre-1992)'/exp OR Zambia/exp OR Zimbabwe/exp |
| **5.** | #1 AND #2 AND #3 AND #4 |
| **Database: Global Health (Ovid)** | |
| **1.** | "Neoplasms*" OR "neoplas*" OR "Tumor*" OR "Cancer*" OR "Malignan*" |
| **2.** | "Decision making" OR "Clinical Decision-Making" OR "Clinical decision support" |
| **3.** | "Palliative care" OR "Maintenance Chemotherapy" OR "hormone replacement therapy" OR "Treatment*" OR "intervention*" OR "chemotherap*" OR "cancer therap*" OR "palliative chemotherap*" OR "Metronomic Administration*" OR "palliative radiation" OR "palliative surger*" OR "Palliative Treatment*" OR "Palliative Therap*" OR "Bone Marrow Graft*" OR "hormone therap*" OR "drug maintenance" OR "maintenance treatment" OR "maintenance therap*" OR "bone marrow transplant*" OR "hormonal therap*" OR "hormone treatment*" |
| **4.** | "developing country" OR "developing countries" OR "developing nation" OR "developing nations" OR "developing population" OR "developing populations" OR "developing world" OR "less developed country" OR "less developed countries" OR "less developed nation" OR "less developed nations" OR "less developed population" OR "less developed populations" OR "less developed world" OR "lesser developed country" OR "lesser developed countries" OR "lesser developed nation" OR "lesser developed nations" OR "lesser developed population" OR "lesser developed populations" OR "lesser developed world" OR "under developed country" OR "under developed countries" OR "under developed nation" OR "under developed nations" OR "under developed population" OR "under developed populations" OR "under developed world" OR "underdeveloped country" OR "underdeveloped countries" OR "underdeveloped nation" OR "underdeveloped nations" OR "underdeveloped population" OR "underdeveloped populations" OR "underdeveloped world" OR "middle income country" OR "middle income countries" OR "middle income nation" OR "middle income nations" OR "middle income population" OR "middle income populations" OR "low income country" OR "low income countries" OR "low income nation" OR "low income nations" OR "low income population" OR "low income populations" OR "lower income country" OR "lower income countries" OR "lower income nation" OR "lower income nations" OR "lower income population" OR "lower income populations" OR "underserved country" OR "underserved countries" OR "underserved nation" OR "underserved nations" OR "underserved population" OR "underserved populations" OR "underserved world" OR "under served country" OR "under served countries" OR "under served nation" OR "under served nations" OR "under served population" OR "under served populations" OR "under served world" OR "deprived country" OR "deprived countries" OR "deprived nation" OR "deprived nations" OR "deprived population" OR "deprived populations" OR "deprived world" OR "poor country" OR "poor countries" OR "poor nation" OR "poor nations" OR "poor population" OR "poor populations" OR "poor world" OR "poorer country" OR "poorer countries" OR "poorer nation" OR "poorer nations" OR "poorer population" OR "poorer populations" OR "poorer world" OR "developing economy" OR "developing economies" OR "less developed economy" OR "less developed economies" OR "lesser developed economy" OR "lesser developed economies" OR "under developed economy" OR "under developed economies" OR "underdeveloped economy" OR "underdeveloped economies" OR "middle income economy" OR "middle income economies" OR "low income economy" OR "low income economies" OR "lower income economy" OR "lower income economies" OR "low gdp" OR "low gnp" OR "low gross domestic" OR "low gross national" OR "lower gdp" OR "lower gnp" OR "lower gross domestic" OR "lower gross national" OR lmic OR lmics OR "third world" OR "lami country" OR "lami countries" OR "transitional country" OR "transitional countries").tw. OR Africa OR Asia OR Caribbean OR "West Indies" OR "South America" OR "Latin America" OR "Central America" OR "Atlantic Islands" OR "Commonwealth of Independent States" OR "Pacific Islands" OR "Indian Ocean Islands" OR "Eastern Europe" OR "Baltic States" OR Afghanistan OR Albania OR Algeria OR Angola OR Antigua OR Barbuda OR Argentina OR Armenia OR Armenian OR Aruba OR Azerbaijan OR Bahrain OR Bangladesh OR Barbados OR Benin OR Byelarus OR Byelorussian OR Belarus OR Belorussian OR Belorussia OR Belize OR Bhutan OR Bolivia OR Bosnia OR Herzegovina OR Hercegovina OR Botswana OR Brasil OR Brazil OR Bulgaria OR "Burkina Faso" OR "Burkina Fasso" OR "Upper Volta" OR Burundi OR Urundi OR Cambodia OR "Khmer Republic" OR Kampuchea OR Cameroon OR Cameroons OR Cameron OR Camerons OR "Cape Verde" OR "Cabo Verde" OR "Central African Republic" OR Chad OR Chile OR China OR Colombia OR Comoros OR "Comoro Islands" OR Comores OR Mayotte OR Congo OR Zaire OR "Costa Rica" OR "Cote d'Ivoire" OR "Ivory Coast" OR Croatia OR Cuba OR Cyprus OR Czechoslovakia OR "Czech Republic" OR Slovakia OR "Slovak Republic" OR Djibouti OR "French Somaliland" OR Dominica OR "Dominican Republic" OR "East Timor" OR "East Timur" OR "Timor Leste" OR Ecuador OR Egypt OR "United Arab Republic" OR "El Salvador" OR Eritrea OR Estonia OR Eswatini OR Ethiopia OR Fiji OR Gabon OR "Gabonese Republic" OR Gambia OR Gaza OR "Georgia Republic" OR "Georgian Republic" OR Ghana OR "Gold Coast" OR Greece OR Grenada OR Guatemala OR Guinea OR Guam OR Guiana OR Guyana OR Haiti OR Honduras OR Hungary OR India OR Maldives OR Indonesia OR Iran OR Iraq OR "Isle of Man" OR Jamaica OR Jordan OR Kazakhstan OR Kazakh OR Kenya OR Kiribati OR Nauru OR Korea OR Kosovo OR Kyrgyzstan OR Kirghizia OR "Kyrgyz Republic" OR Kirghiz OR Kirgizstan OR "Lao PDR" OR Laos OR Latvia OR Lebanon OR Lesotho OR Basutoland OR Liberia OR Libya OR Lithuania).tw. OR (Macedonia OR Madagascar OR "Malagasy Republic" OR Malaysia OR Malaya OR Malay OR Sabah OR Sarawak OR Malawi OR Nyasaland OR Mali OR Malta OR "Marshall Islands" OR Mauritania OR Mauritius OR "Agalega Islands" OR Melanesia OR Mexico OR Micronesia OR "Middle East" OR Moldova OR Moldovia OR Moldovian OR Mongolia OR Montenegro OR Morocco OR Ifni OR Mozambique OR Myanmar OR Myanma OR Burma OR Namibia OR Nepal OR "Netherlands Antilles" OR "New Caledonia" OR Nicaragua OR Niger OR Nigeria OR "Northern Mariana Islands" OR Oman OR Muscat OR Pakistan OR Palau OR Palestine OR Panama OR Paraguay OR Peru OR Philippines OR Philipines OR Phillipines OR Phillippines OR Poland OR Portugal OR "Puerto Rico" OR Romania OR Rumania OR Roumania OR Russia OR Russian OR Rwanda OR Ruanda OR "Saint Kitts" OR "St Kitts" OR Nevis OR "Saint Lucia" OR "St Lucia" OR "Saint Vincent" OR "St Vincent" OR Grenadines OR Samoa OR "Samoan Islands" OR "Navigator Island" OR "Navigator Islands" OR "Sao Tome" OR "Saudi Arabia" OR Senegal OR Serbia OR Montenegro OR Seychelles OR "Sierra Leone" OR Slovenia OR "Sri Lanka" OR Ceylon OR "Solomon Islands" OR Somalia OR Sudan OR Suriname OR Surinam OR Swaziland OR Syria OR Syrian OR Tajikistan OR Tadzhikistan OR Tadjikistan OR Tadzhik OR Tanzania OR Thailand OR Togo OR "Togolese Republic" OR Tonga OR Trinidad OR Tobago OR Tunisia OR Turkey OR Turkmenistan OR Turkmen OR Tuvalu OR Uganda OR Ukraine OR Uruguay OR USSR OR "Soviet Union" OR "Union of Soviet Socialist Republics" OR Uzbekistan OR Uzbek OR Vanuatu OR "New Hebrides" OR Venezuela OR Vietnam OR "Viet Nam" OR "West Bank" OR Yemen OR Yugoslavia OR Zambia OR Zimbabwe OR Rhodesia).tw. OR exp developing countries/ OR exp algeria/ OR exp angola/ OR exp anguilla island/ OR exp "antigua and barbuda"/ OR exp argentina/ OR exp aruba/ OR exp bahamas/ OR exp bahrain/ OR exp barbados/ OR exp belize/ OR exp bermuda/ OR exp bolivia/ OR exp bonaire/ OR exp brazil/ OR exp british virgin islands/ OR exp brunei darussalam/ OR exp cameroon/ OR exp cayman islands/ OR exp chile/ OR exp china/ OR exp "christmas island (indian ocean)"/ OR exp cocos islands/ OR exp colombia/ OR exp congo/ OR exp cook islands/ OR exp costa rica/ OR exp cote d'ivoire/ OR exp crozet islands/ OR exp cuba/ OR exp curacao/ OR exp cyprus/ OR exp dominica/ OR exp dominican republic/ OR exp easter island/ OR exp ecuador/ OR exp egypt/ OR exp el salvador/ OR exp falkland islands/ OR exp "federated states of micronesia"/ OR exp fiji/ OR exp french guiana/ OR exp gabon/ OR exp gambier islands/ OR exp ghana/ OR exp grenada/ OR exp guadeloupe/ OR exp guam/ OR exp guatemala/ OR exp guyana/ OR exp honduras/ OR exp india/ OR exp indonesia/ OR exp iran/ OR exp iraq/ OR exp jamaica/ OR exp jordan/ OR exp kenya/ OR exp kerguelen archipelago/ OR exp korea democratic people's republic/ OR exp korea republic/ OR exp kuwait/ OR exp least developed countries/ OR exp lebanon/ OR exp libya/ OR exp malaysia/ OR exp marquesas islands/ OR exp marshall islands/ OR exp martinique/ OR exp mauritius/ OR exp mayotte/ OR exp mexico/ OR exp midway islands/ OR exp mongolia/ OR exp montserrat/ OR exp morocco/ OR exp namibia/ OR exp new britain/ OR exp new caledonia/ OR exp new ireland/ OR exp nicaragua/ OR exp nigeria/ OR exp niue/ OR exp northern mariana islands/ OR exp oman/ OR exp pakistan/ OR exp palau/ OR exp panama/ OR exp papua new guinea/ OR exp paraguay/ OR exp peru/ OR exp philippines/ OR exp puerto rico/ OR exp qatar/ OR exp reunion/ OR exp saba/ OR exp saint helena/ OR exp "saint kitts and nevis"/ OR exp saint lucia/ OR exp "saint vincent and the grenadines"/ OR exp saudi arabia/ OR exp senegal/ OR exp seychelles/ OR exp singapore/ OR exp south africa/ OR exp sri lanka/ OR exp suriname/ OR exp swaziland/ OR exp syria/ OR exp tahiti/ OR exp thailand/ OR exp tokelau/ OR exp tonga/ OR exp "trinidad and tobago"/ OR exp tuamotu/ OR exp tubuai islands/ OR exp tunisia/ OR exp turkey/ OR exp "turks and caicos islands"/ OR exp united arab emirates/ OR exp uruguay/ OR exp venezuela/ OR exp vietnam/ OR exp "wallis and futuna"/ OR exp western sahara/ OR exp zimbabwe/ OR exp threshold countries/ OR exp algeria/ OR exp argentina/ OR exp brazil/ OR exp chile/ OR exp costa rica/ OR exp cyprus/ OR exp dominican republic/ OR exp ecuador/ OR exp iran/ OR exp iraq/ OR exp jamaica/ OR exp korea republic/ OR exp lebanon/ OR exp malaysia/ OR exp mexico/ OR exp nicaragua/ OR exp panama/ OR exp singapore/ OR exp south africa/ OR exp syria/ OR exp "trinidad and tobago"/ OR exp tunisia/ OR exp uruguay/ OR exp venezuela/ OR exp yemen/ OR exp least developed countries/ OR exp afghanistan/ OR exp american samoa/ OR exp bangladesh/ OR exp benin/ OR exp bhutan/ OR exp botswana/ OR exp burkina faso/ OR exp burundi/ OR exp cambodia/ OR exp cape verde/ OR exp central african republic/ OR exp chad/ OR exp comoros/ OR exp congo democratic republic/ OR exp djibouti/ OR exp equatorial guinea/ OR exp eritrea/ OR exp ethiopia/ OR exp gambia/ OR exp guinea/ OR exp guinea-bissau/ OR exp haiti/ OR exp kiribati/ OR exp laos/ OR exp lesotho/ OR exp liberia/ OR exp madagascar/ OR exp malawi/ OR exp maldives/ OR exp mali/ OR exp mauritania/ OR exp mozambique/ OR exp myanmar/ OR exp nepal/ OR exp niger/ OR exp rwanda/ OR exp samoa/ OR exp "sao tome and principe"/ OR exp sierra leone/ OR exp solomon islands/ OR exp somalia/ OR exp sudan/ OR exp tanzania/ OR exp togo/ OR exp tuvalu/ OR exp uganda/ OR exp vanuatu/ OR exp yemen/ OR exp zambia/ OR africa/ OR "africa south of sahara"/ OR north africa/ OR west africa/ OR central africa/ OR east africa/ OR southern africa/ OR south east asia/ OR agrarian countries/ OR asia/ OR west asia/ OR east asia/ OR south asia/ OR central asia/ OR caribbean/ OR central america/ OR colonies/ OR latin america/ OR oceania/ OR south america/ OR pacific islands/ OR indian ocean islands/ OR central europe/ OR albania/ OR armenia/ OR azerbaijan/ OR belarus/ OR bosnia-hercegovina/ OR bulgaria/ OR exp china/ OR croatia/ OR czechoslovakia/ OR czech republic/ OR slovakia/ OR east timor/ OR estonia/ OR "republic of georgia"/ OR exp greece/ OR hungary/ OR kazakhstan/ OR kosovo/ OR kyrgyzstan/ OR latvia/ OR lithuania/ OR "republic of macedonia"/ OR malta/ OR exp melanesia/ OR exp micronesia/ OR middle east/ OR moldova/ OR montenegro/ OR exp netherlands antilles/ OR exp palestine/ OR poland/or exp portugal/ OR romania/ OR exp russia/ OR "serbia and montenegro"/ OR montenegro/ OR exp serbia/ OR slovenia/ OR tajikistan/ OR turkmenistan/ OR ukraine/ OR exp ussr/ OR uzbekistan/ OR yugoslavia/ |
| **5.** | #1 AND #2 AND #3 AND #4 |
| **Database: Global Index Medicus** | |
| **1.** | "Neoplasms*" OR "neoplas*" OR "Tumor*" OR "Cancer*" OR "Malignan*" |
| **2.** | "Decision making" OR "Clinical Decision-Making" OR "Clinical decision support" |
| **3.** | "Palliative care" OR "Maintenance Chemotherapy" OR "hormone replacement therapy" OR "Treatment*" OR "intervention*" OR "chemotherap*" OR "cancer therap*" OR "palliative chemotherap*" OR "Metronomic Administration*" OR "palliative radiation" OR "palliative surger*" OR "Palliative Treatment*" OR "Palliative Therap*" OR "Bone Marrow Graft*" OR "hormone therap*" OR "drug maintenance" OR "maintenance treatment" OR "maintenance therap*" OR "bone marrow transplant*" OR "hormonal therap*" OR "hormone treatment*" |
| **4.** | #1 AND #2 AND #3 |
| **Database search: PubMed** | |
| **1.** | "Neoplasms"[mh] OR "neoplas*"[tw] OR "Tumor*"[tw] OR "Cancer*"[tw] OR "Malignan*"[tw] |
| **2.** | "Decision making"[mh] OR "Decision Making, Shared"[mh] OR "Clinical Decision-Making"[mh] OR "Clinical decision support"[tw] OR "decision making"[tw] |
| **3.** | "Administration, Metronomic"[mesh] OR "Palliative care"[mesh] OR "Maintenance Chemotherapy"[mh] OR "Bone Marrow Transplantation"[mh] OR "hormone replacement therapy"[mh] OR "Treatment*"[tw] OR "intervention*"[tw] OR "chemotherap*"[tw] OR "cancer therap*"[tw] OR "palliative chemotherap*"[tw] OR "Metronomic Administration*"[tw] OR "palliative radiation"[tw] OR "palliative surger*"[tw] OR "Palliative Treatment*"[tw] OR "Palliative Therap*"[tw] OR "palliative care"[tw] OR "Maintenance Chemotherap*"[tw] OR "Bone Marrow Graft*"[tw] OR "hormone therap*"[tw] OR "drug maintenance"[tw] OR "maintenance treatment"[tw] OR "maintenance therap*"[tw] OR "bone marrow graft*"[tw] OR "bone marrow transplant*"[tw] OR "hormonal therap*"[tw] OR "hormone treatment*"[tw] |
| **4.** | afghanistan[tw] OR albania[tw] OR algeria[tw] OR american samoa[tw] OR angola[tw] OR antigua[tw] OR barbuda[tw] OR argentina[tw] OR armenia[tw] OR armenian[tw] OR aruba[tw] OR azerbaijan[tw] OR bahrain[tw] OR bangladesh[tw] OR barbados[tw] OR belarus[tw] OR byelarus[tw] OR belorussia[tw] OR byelorussian[tw] OR belize[tw] OR british honduras[tw] OR benin[tw] OR dahomey[tw] OR bhutan[tw] OR bolivia[tw] OR bosnia[tw] OR herzegovina[tw] OR botswana[tw] OR bechuanaland[tw] OR brazil[tw] OR brasil[tw] OR bulgaria[tw] OR burkina faso[tw] OR burkina fasso[tw] OR upper volta[tw] OR burundi[tw] OR urundi[tw] OR cabo verde[tw] OR cape verde[tw] OR cambodia[tw] OR kampuchea[tw] OR khmer republic[tw] OR cameroon[tw] OR cameron[tw] OR cameroun[tw] OR central african republic[tw] OR ubangi shari[tw] OR chad[tw] OR chile[tw] OR china[tw] OR colombia[tw] OR comoros[tw] OR comoro islands[tw] OR mayotte[tw] OR congo[tw] OR zaire[tw] OR costa rica[tw] OR cote d'ivoire[tw] OR cote d'ivoire[tw] OR cote d'ivoire[tw] OR ivory coast[tw] OR croatia[tw] OR cuba[tw] OR cyprus[tw] OR czech republic[tw] OR czechoslovakia[tw] OR djibouti[tw] OR french somaliland[tw] OR dominica[tw] OR dominican republic[tw] OR ecuador[tw] OR egypt[tw] OR united arab republic[tw] OR el salvador[tw] OR equatorial guinea[tw] OR spanish guinea[tw] OR eritrea[tw] OR estonia[tw] OR eswatini[tw] OR swaziland[tw] OR ethiopia[tw] OR fiji[tw] OR gabon[tw] OR gabonese republic[tw] OR gambia[tw] OR georgia[tw] OR georgian[tw] OR ghana[tw] OR gold coast[tw] OR gibraltar[tw] OR greece[tw] OR grenada[tw] OR guam[tw] OR guatemala[tw] OR guinea[tw] OR guyana[tw] OR guiana[tw] OR haiti[tw] OR hispaniola[tw] OR honduras[tw] OR hungary[tw] OR india[tw] OR indonesia[tw] OR timor[tw] OR iran[tw] OR iraq[tw] OR isle of man[tw] OR jamaica[tw] OR jordan[tw] OR kazakhstan[tw] OR kazakh[tw] OR kenya[tw] OR korea[tw] OR kosovo[tw] OR kyrgyzstan[tw] OR kirghizia[tw] OR kirgizstan[tw] OR kyrgyz republic[tw] OR kirghiz[tw] OR laos[tw] OR lao pdr[tw] OR lao people's democratic republic[tw] OR latvia[tw] OR lebanon[tw] OR lesotho[tw] OR basutoland[tw] OR liberia[tw] OR libya[tw] OR libyan arab jamahiriya[tw] OR lithuania[tw] OR macau[tw] OR macao[tw] OR macedonia[tw] OR madagascar[tw] OR malagasy republic[tw] OR malawi[tw] OR nyasaland[tw] OR malaysia[tw] OR maldives[tw] OR indian ocean[tw] OR mali[tw] OR malta[tw] OR micronesia[tw] OR kiribati[tw] OR marshall islands[tw] OR nauru[tw] OR northern mariana islands[tw] OR palau[tw] OR tuvalu[tw] OR mauritania[tw] OR mauritius[tw] OR mexico[tw] OR moldova[tw] OR moldovian[tw] OR mongolia[tw] OR montenegro[tw] OR morocco[tw] OR ifni[tw] OR mozambique[tw] OR portuguese east africa[tw] OR myanmar[tw] OR burma[tw] OR namibia[tw] OR nepal[tw] OR netherlands antilles[tw] OR nicaragua[tw] OR niger[tw] OR nigeria[tw] OR oman[tw] OR muscat[tw] OR pakistan[tw] OR panama[tw] OR papua new guinea[tw] OR paraguay[tw] OR peru[tw] OR philippines[tw] OR philipines[tw] OR phillipines[tw] OR phillippines[tw] OR poland[tw] OR polish people's republic[tw] OR portugal[tw] OR portuguese republic[tw] OR puerto rico[tw] OR romania[tw] OR russia[tw] OR russian federation[tw] OR ussr[tw] OR soviet union[tw] OR union of soviet socialist republics[tw] OR rwanda[tw] OR ruanda[tw] OR samoa[tw] OR pacific islands[tw] OR polynesia[tw] OR samoan islands[tw] OR sao tome and principe[tw] OR saudi arabia[tw] OR senegal[tw] OR serbia[tw] OR seychelles[tw] OR sierra leone[tw] OR slovakia[tw] OR slovak republic[tw] OR slovenia[tw] OR melanesia[tw] OR solomon island[tw] OR solomon islands[tw] OR norfolk island[tw] OR somalia[tw] OR south africa[tw] OR south sudan[tw] OR sri lanka[tw] OR ceylon[tw] OR saint kitts and nevis[tw] OR st kitts and nevis[tw] OR saint lucia[tw] OR st lucia[tw] OR saint vincent[tw] OR st vincent[tw] OR grenadines[tw] OR sudan[tw] OR suriname[tw] OR surinam[tw] OR syria[tw] OR syrian arab republic[tw] OR tajikistan[tw] OR tadjikistan[tw] OR tadzhikistan[tw] OR tadzhik[tw] OR tanzania[tw] OR tanganyika[tw] OR thailand[tw] OR siam[tw] OR timor leste[tw] OR east timor[tw] OR togo[tw] OR togolese republic[tw] OR tonga[tw] OR trinidad[tw] OR tobago[tw] OR tunisia[tw] OR turkey[tw] OR turkmenistan[tw] OR turkmen[tw] OR uganda[tw] OR ukraine[tw] OR uruguay[tw] OR uzbekistan[tw] OR uzbek[tw] OR vanuatu[tw] OR new hebrides[tw] OR venezuela[tw] OR vietnam[tw] OR viet nam[tw] OR middle east[tw] OR west bank[tw] OR gaza[tw] OR palestine[tw] OR yemen[tw] OR yugoslavia[tw] OR zambia[tw] OR zimbabwe[tw] OR northern rhodesia[tw] OR global south[tw] OR africa south of the sahara[tw] OR sub saharan africa[tw] OR subsaharan africa[tw] OR central africa[tw] OR north africa[tw] OR northern africa[tw] OR magreb[tw] OR maghrib[tw] OR sahara[tw] OR southern africa[tw] OR east africa[tw] OR eastern africa[tw] OR west africa[tw] OR western africa[tw] OR west indies[tw] OR indian ocean islands[tw] OR caribbean[tw] OR central america[tw] OR latin america[tw] OR south america[tw] OR central asia[tw] OR north asia[tw] OR northern asia[tw] OR southeastern asia[tw] OR south eastern asia[tw] OR southeast asia[tw] OR south east asia[tw] OR western asia[tw] OR east europe[tw] OR eastern europe[tw] OR developing country[tw] OR developing countries[tw] OR developing nation[tw] OR developing nations[tw] OR developing population[tw] OR developing populations[tw] OR developing world[tw] OR less developed country[tw] OR less developed countries[tw] OR less developed nation[tw] OR less developed nations[tw] OR less developed world[tw] OR lesser developed countries[tw] OR lesser developed nations[tw] OR under developed country[tw] OR under developed countries[tw] OR under developed nations[tw] OR under developed world[tw] OR underdeveloped country[tw] OR underdeveloped countries[tw] OR underdeveloped nation[tw] OR underdeveloped nations[tw] OR underdeveloped population[tw] OR underdeveloped populations[tw] OR underdeveloped world[tw] OR middle income country[tw] OR middle income countries[tw] OR middle income nation[tw] OR middle income nations[tw] OR middle income population[tw] OR middle income populations[tw] OR low income country[tw] OR low income countries[tw] OR low income nation[tw] OR low income nations[tw] OR low income population[tw] OR low income populations[tw] OR lower income country[tw] OR lower income countries[tw] OR lower income nations[tw] OR lower income population[tw] OR lower income populations[tw] OR underserved countries[tw] OR underserved nations[tw] OR underserved population[tw] OR underserved populations[tw] OR under served population[tw] OR under served populations[tw] OR deprived countries[tw] OR deprived population[tw] OR deprived populations[tw] OR poor country[tw] OR poor countries[tw] OR poor nation[tw] OR poor nations[tw] OR poor population[tw] OR poor populations[tw] OR poor world[tw] OR poorer countries[tw] OR poorer nations[tw] OR poorer population[tw] OR poorer populations[tw] OR developing economy[tw] OR developing economies[tw] OR less developed economy[tw] OR less developed economies[tw] OR underdeveloped economies[tw] OR middle income economy[tw] OR middle income economies[tw] OR low income economy[tw] OR low income economies[tw] OR lower income economies[tw] OR low gdp[tw] OR low gnp[tw] OR low gross domestic[tw] OR low gross national[tw] OR lower gdp[tw] OR lower gross domestic[tw] OR lmic[tw] OR lmics[tw] OR third world[tw] OR lami country[tw] OR lami countries[tw] OR transitional country[tw] OR transitional countries[tw] OR emerging economies[tw] OR emerging nation[tw] OR emerging nations[tw] OR afghanistan[mh] OR albania[mh] OR algeria[mh] OR american samoa[mh] OR angola[mh] OR antigua and barbuda[mh] OR argentina[mh] OR armenia[mh] OR aruba[mh] OR azerbaijan[mh] OR bahrain[mh] OR bangladesh[mh] OR barbados[mh] OR republic of belarus[mh] OR belize[mh] OR benin[mh] OR bhutan[mh] OR bolivia[mh] OR bosnia and herzegovina[mh] OR botswana[mh] OR brazil[mh] OR bulgaria[mh] OR burkina faso[mh] OR burundi[mh] OR cabo verde[mh] OR cambodia[mh] OR cameroon[mh] OR central african republic[mh] OR chad[mh] OR chile[mh] OR china[mh] OR colombia[mh] OR comoros[mh] OR democratic republic of the congo[mh] OR congo[mh] OR costa rica[mh] OR cote d'ivoire[mh] OR croatia[mh] OR cuba[mh] OR cyprus[mh] OR czech republic[mh] OR djibouti[mh] OR dominica[mh] OR dominican republic[mh] OR ecuador[mh] OR egypt[mh] OR el salvador[mh] OR equatorial guinea[mh] OR eritrea[mh] OR estonia[mh] OR eswatini[mh] OR ethiopia[mh] OR fiji[mh] OR gabon[mh] OR gambia[mh] OR "georgia republic"[mh] OR ghana[mh] OR gibraltar[mh] OR greece[mh] OR grenada[mh] OR guam[mh] OR guatemala[mh] OR guinea[mh] OR guinea-bissau[mh] OR guyana[mh] OR haiti[mh] OR honduras[mh] OR hungary[mh] OR india[mh] OR indonesia[mh] OR iran[mh] OR iraq[mh] OR jamaica[mh] OR jordan[mh] OR kazakhstan[mh] OR kenya[mh] OR democratic people's republic of korea[mh] OR republic of korea[mh] OR kosovo[mh] OR kyrgyzstan[mh] OR laos[mh] OR latvia[mh] OR lebanon[mh] OR lesotho[mh] OR liberia[mh] OR libya[mh] OR lithuania[mh] OR macau[mh] OR republic of north macedonia[mh] OR madagascar[mh] OR malawi[mh] OR malaysia[mh] OR indian ocean islands[mh] OR mali[mh] OR malta[mh] OR micronesia[mh] OR palau[mh] OR mauritania[mh] OR mauritius[mh] OR mexico[mh] OR moldova[mh] OR mongolia[mh] OR montenegro[mh] OR morocco[mh] OR mozambique[mh] OR myanmar[mh] OR namibia[mh] OR nepal[mh] OR netherlands antilles[mh] OR nicaragua[mh] OR niger[mh] OR nigeria[mh] OR oman[mh] OR pakistan[mh] OR panama[mh] OR papua new guinea[mh] OR paraguay[mh] OR peru[mh] OR philippines[mh] OR poland[mh] OR portugal[mh] OR puerto rico[mh] OR romania[mh] OR russia[mh] OR rwanda[mh] OR samoa[mh] OR sao tome and principe[mh] OR saudi arabia[mh] OR senegal[mh] OR serbia[mh] OR seychelles[mh] OR sierra leone[mh] OR slovakia[mh] OR slovenia[mh] OR melanesia[mh] OR somalia[mh] OR south africa[mh] OR south sudan[mh] OR sri lanka[mh] OR saint kitts and nevis[mh] OR saint lucia[mh] OR saint vincent and the grenadines[mh] OR sudan[mh] OR suriname[mh] OR syria[mh] OR tajikistan[mh] OR tanzania[mh] OR thailand[mh] OR timor-leste[mh] OR togo[mh] OR tonga[mh] OR trinidad and tobago[mh] OR tunisia[mh] OR turkey[mh] OR turkmenistan[mh] OR uganda[mh] OR ukraine[mh] OR uruguay[mh] OR uzbekistan[mh] OR vanuatu[mh] OR venezuela[mh] OR vietnam[mh] OR middle east[mh] OR yemen[mh] OR yugoslavia[mh] OR zambia[mh] OR zimbabwe[mh] OR africa south of the sahara[mh] OR africa, central[mh] OR africa, northern[mh] OR africa, southern[mh] OR africa, eastern[mh] OR africa, western[mh] OR west indies[mh] OR indian ocean islands[mh] OR caribbean region[mh] OR central america[mh] OR latin america[mh] OR south america[mh] OR asia, central[mh] OR asia, northern[mh] OR asia, southeastern[mh] OR asia, western[mh] OR europe, eastern[mh] OR developing countries[mh] |
| **5.** | #1 AND #2 AND #3 AND #4 |
| **Database: Scopus** | |
| **1.** | TITLE-ABS-KEY "Neoplasm*" OR "neoplastic" OR "Tumor*" OR "Cancer*" OR "Malignancy" OR "malignancies" |
| **2.** | TITLE-ABS-KEY "Decision making" OR "Clinical Decision-Making" OR "Clinical decision support" |
| **3.** | TITLE-ABS-KEY "Palliative care" OR "Maintenance Chemotherapy" OR "Bone Marrow Transplantation" OR "hormone replacement therapy" OR "Treatment*" OR "intervention*" OR "chemotherap*" OR "cancer therap*" OR "palliative radiation" OR "palliative surger*" OR "Palliative Treatment*" OR "Palliative Therap*" OR "Bone Marrow Graft*" OR "hormone therap*" OR "drug maintenance" OR "maintenance treatment" OR "maintenance therap*" OR "bone marrow transplant*" OR "hormonal therap*" OR "hormone treatment*" |
| **4.** | TITLE-ABS-KEY "developing country" OR "developing countries" OR "developing nation" OR "developing nations" OR "developing population" OR "developing populations" OR "developing world" OR "less developed country" OR "less developed countries" OR "less developed nation" OR "less developed nations" OR "less developed population" OR "less developed populations" OR "less developed world" OR "lesser developed country" OR "lesser developed countries" OR "lesser developed nation" OR "lesser developed nations" OR "lesser developed population" OR "lesser developed populations" OR "lesser developed world" OR "under developed country" OR "under developed countries" OR "under developed nation" OR "under developed nations" OR "under developed population" OR "under developed populations" OR "under developed world" OR "underdeveloped country" OR "underdeveloped countries" OR "underdeveloped nation" OR "underdeveloped nations" OR "underdeveloped population" OR "underdeveloped populations" OR "underdeveloped world" OR "middle income country" OR "middle income countries" OR "middle income nation" OR "middle income nations" OR "middle income population" OR "middle income populations" OR "low income country" OR "low income countries" OR "low income nation" OR "low income nations" OR "low income population" OR "low income populations" OR "lower income country" OR "lower income countries" OR "lower income nation" OR "lower income nations" OR "lower income population" OR "lower income populations" OR "underserved country" OR "underserved countries" OR "underserved nation" OR "underserved nations" OR "underserved population" OR "underserved populations" OR "underserved world" OR "under served country" OR "under served countries" OR "under served nation" OR "under served nations" OR "under served population" OR "under served populations" OR "under served world" OR "deprived country" OR "deprived countries" OR "deprived nation" OR "deprived nations" OR "deprived population" OR "deprived populations" OR "deprived world" OR "poor country" OR "poor countries" OR "poor nation" OR "poor nations" OR "poor population" OR "poor populations" OR "poor world" OR "poorer country" OR "poorer countries" OR "poorer nation" OR "poorer nations" OR "poorer population" OR "poorer populations" OR "poorer world" OR "developing economy" OR "developing economies" OR "less developed economy" OR "less developed economies" OR "lesser developed economy" OR "lesser developed economies" OR "under developed economy" OR "under developed economies" OR "underdeveloped economy" OR "underdeveloped economies" OR "middle income economy" OR "middle income economies" OR "low income economy" OR "low income economies" OR "lower income economy" OR "lower income economies" OR "low gdp" OR "low gnp" OR "low gross domestic" OR "low gross national" OR "lower gdp" OR "lower gnp" OR "lower gross domestic" OR "lower gross national" OR "lmic" OR "lmics" OR "third world" OR "lami country" OR "lami countries" OR "transitional country" OR "transitional countries" OR "Africa" OR "Asia" OR "Caribbean" OR "West Indies" OR "South America" OR "Latin America" OR "Central America" OR "Atlantic Islands" OR "Commonwealth of Independent States" OR "Pacific Islands" OR "Indian Ocean Islands" OR "Eastern Europe" OR "Baltic States" OR "Afghanistan" OR "Albania" OR "Algeria" OR "Angola" OR "Antigua" OR "Barbuda" OR "Argentina" OR "Armenia" OR "Armenian" OR "Aruba" OR "Azerbaijan" OR "Bahrain" OR "Bangladesh" OR "Barbados" OR "Benin" OR "Byelarus" OR "Byelorussian" OR "Belarus" OR "Belorussian" OR "Belorussia" OR "Belize" OR "Bhutan" OR "Bolivia" OR "Bosnia" OR "Herzegovina" OR "Hercegovina" OR "Botswana" OR "Brasil" OR "Brazil" OR "Bulgaria" OR "Burkina Faso" OR "Burkina Fasso" OR "Upper Volta" OR "Burundi" OR "Urundi" OR "Cambodia" OR "Khmer Republic" OR "Kampuchea" OR "Cameroon" OR "Cameroons" OR "Cameron" OR "Camerons" OR "Cape Verde" OR "Cabo Verde" OR "Central African Republic" OR "Chad" OR "Chile" OR "China" OR "Colombia" OR "Comoros" OR "Comoro Islands" OR "Comores" OR "Mayotte" OR "Congo" OR "Zaire" OR "Costa Rica" OR "Cote d'Ivoire" OR "Ivory Coast" OR "Croatia" OR "Cuba" OR "Cyprus" OR "Czechoslovakia" OR "Czech Republic" OR "Slovakia" OR "Slovak Republic" OR "Djibouti" OR "French Somaliland" OR "Dominica" OR "Dominican Republic" OR "East Timor" OR "East Timur" OR "Timor Leste" OR "Ecuador" OR "Egypt" OR "United Arab Republic" OR "El Salvador" OR "Eritrea" OR "Estonia" OR “Eswatini” OR "Ethiopia" OR "Fiji" OR "Gabon" OR "Gabonese Republic" OR "Gambia" OR "Gaza" OR "Georgia Republic" OR "Georgian Republic" OR "Ghana" OR "Gold Coast" OR "Greece" OR "Grenada" OR "Guatemala" OR "Guinea" OR "Guam" OR "Guiana" OR "Guyana" OR "Haiti" OR "Honduras" OR "Hungary" OR "India" OR "Maldives" OR "Indonesia" OR "Iran" OR "Iraq" OR "Isle of Man" OR "Jamaica" OR "Jordan" OR "Kazakhstan" OR "Kazakh" OR "Kenya" OR "Kiribati" OR "Nauru" OR "Korea" OR "Kosovo" OR "Kyrgyzstan" OR "Kirghizia" OR "Kyrgyz Republic" OR "Kirghiz" OR "Kirgizstan" OR "Lao PDR" OR "Laos" OR "Latvia" OR "Lebanon" OR "Lesotho" OR "Basutoland" OR "Liberia" OR "Libya" OR "Lithuania" OR "Macedonia" OR "Madagascar" OR "Malagasy Republic" OR "Malaysia" OR "Malaya" OR "Malay" OR "Sabah" OR "Sarawak" OR "Malawi" OR "Nyasaland" OR "Mali" OR "Malta" OR "Marshall Islands" OR "Mauritania" OR "Mauritius" OR "Agalega Islands" OR "Melanesia" OR "Mexico" OR "Micronesia" OR "Middle East" OR "Moldova" OR "Moldovia" OR "Moldovian" OR "Mongolia" OR "Montenegro" OR "Morocco" OR "Ifni" OR "Mozambique" OR "Myanmar" OR "Myanma" OR "Burma" OR "Namibia" OR "Nepal" OR "Netherlands Antilles" OR "New Caledonia" OR "Nicaragua" OR "Niger" OR "Nigeria" OR "Northern Mariana Islands" OR "Oman" OR "Muscat" OR "Pakistan" OR "Palau" OR "Palestine" OR "Panama" OR "Paraguay" OR "Peru" OR "Philippines" OR "Philipines" OR "Phillipines" OR "Phillippines" OR "Poland" OR "Portugal" OR "Puerto Rico" OR "Romania" OR "Rumania" OR "Roumania" OR "Russia" OR "Russian" OR "Rwanda" OR "Ruanda" OR "Saint Kitts" OR "St Kitts" OR "Nevis" OR "Saint Lucia" OR "St Lucia" OR "Saint Vincent" OR "St Vincent" OR "Grenadines" OR "Samoa" OR "Samoan Islands" OR "Navigator Island" OR "Navigator Islands" OR "Sao Tome" OR "Saudi Arabia" OR "Senegal" OR "Serbia" OR "Montenegro" OR "Seychelles" OR "Sierra Leone" OR "Slovenia" OR "Sri Lanka" OR "Ceylon" OR "Solomon Islands" OR "Somalia" OR "Sudan" OR "Suriname" OR "Surinam" OR "Swaziland" OR "Syria" OR "Syrian" OR "Tajikistan" OR "Tadzhikistan" OR "Tadjikistan" OR "Tadzhik" OR "Tanzania" OR "Thailand" OR "Togo" OR "Togolese Republic" OR "Tonga" OR "Trinidad" OR "Tobago" OR "Tunisia" OR "Turkey" OR "Turkmenistan" OR "Turkmen" OR "Tuvalu" OR "Uganda" OR "Ukraine" OR "Uruguay" OR "USSR" OR "Soviet Union" OR "Union of Soviet Socialist Republics" OR "Uzbekistan" OR "Uzbek" OR "Vanuatu" OR "New Hebrides" OR "Venezuela" OR "Vietnam" OR "Viet Nam" OR "West Bank" OR "Yemen" OR "Yugoslavia" OR "Zambia" OR "Zimbabwe" OR "Rhodesia" |
| **5.** | #1 AND #2 AND #3 AND #4 |
| **Database: Worldwidescience.org** | |
| **1.** | "Neoplasms*" OR "neoplas*" OR "Tumor*" OR "Cancer*" OR "Malignan*" |
| **2.** | "Decision making" OR "Clinical Decision-Making" OR "Clinical decision support" |
| **3.** | "Palliative care" OR "Maintenance Chemotherapy" OR "hormone replacement therapy" OR "Treatment*" OR "intervention*" OR "chemotherap*" OR "cancer therap*" OR "palliative chemotherap*" OR "Metronomic Administration*" OR "palliative radiation" OR "palliative surger*" OR "Palliative Treatment*" OR "Palliative Therap*" OR "Bone Marrow Graft*" OR "hormone therap*" OR "drug maintenance" OR "maintenance treatment" OR "maintenance therap*" OR "bone marrow transplant*" OR "hormonal therap*" OR "hormone treatment*" |

Supplemental Table 2. Study eligibility criteria.

| **Research Question:** The purpose of this scoping review is to evaluate existing literature related to treatment decision-making for patients diagnosed with cancer in low- and middle-income countries and to identify discrete factors that influence decision-making for all involved parties, which may include patients, families and caregivers, healthcare professionals, and community members. | |
| --- | --- |
| **Inclusion** | **Exclusion** |
| - Studies/articles must describe medical decision making at any point during cancer treatment and can be described from any perspective, for example, physician or patient and family - Studies/articles involve patients of all ages (neonate, child, adolescent, or adult) who are diagnosed with any type of cancer - All types of studies/articles can be considered, including original research, reviews, editorials, commentaries, conference presentations - Studies/articles that conducted or reflect the perspective of cancer care in low- and middle-income countries; include studies/articles if they are conducted in both low- and middle-income countries and high-income countries if described together - Studies/articles have no restriction based on date published | - Studies/articles that do not involve any patients diagnosed with cancer - Studies/articles that are solely conducted high-income countries - Studies/articles related to cancer screening that do not also discuss treatment decision-making - Studies/articles related to assessment or staging of cancers, including prognostic factors, imaging techniques, lab-based methods, surgical interventions, genetic testing, or histopathology without also discussing treatment decision-making - Studies/articles focused on specific treatments of cancers without also discussing treatment decision-making - Studies/articles related to decision making only about end-of-life care - Studies/articles related to managing complications of cancer treatment, for example pain, nausea, vomiting, or fever, without also discussing treatment decision-making - Studies/articles describing the role of artificial intelligence or other technologies in making medical treatment decisions - Studies/articles not published in English |

Supplemental Table 3. Definitions of factors influencing cancer treatment decision-making in low- and middle-income countries. Factors are described with associated subfactors, as well as examples of terms used in articles included in this scoping review. Please note, citations here align with the reference list included with the supplemental material and not the main manuscript.

| **Factor** | **Subfactors** | **Additional Examples** |
| --- | --- | --- |
| Personal Belief System/Characteristics  *Includes beliefs/characteristics of all parties (patient, caregiver, multidisciplinary team) involved in decision making* | Motivation, Personal Wishes | Patient/parent preference,^1-6^ desire to recover,^7^ fear of recurrence and metastasis,^2,4,8-10^ physician recommendation that does not factor patient opinion or wishes,^11^ consent,^12^ physician uncertainty aversion,^13^ physician personality traits,^13,14^ physician demographics (age, gender),^13^ attitudes towards surgery,^5^ patient’s number of children,^15^ lactation history,^15^ emotional distress of parent (risk of child suffering),^16^ desire to live,^17,18^ desire to achieve cure,^19^ minimize chances of dying of cancer,^20^ physicians’ unwillingness to change their usual practice^21^ |
| Quality of Life  *Of patient* | Psychological Morbidity,^22^ Emotional Stress | Quality of life,^23^ preference related to mode of treatment administration (for example, avoiding intravenous medications),^9,10,20,24^ willingness to tolerate side effects,^25^ potential for suffering,^26,27^ maintaining female body shape to facilitate future work and life,^2^ influence on marriage and procreation,^2^ influence on marital relationship,^2^ recovery,^2,9^ impact on body image,^4,9^ fear of needing repeat surgery,^10^ patient satisfaction,^4^ satisfaction with cosmetic body image,^4,8^ satisfaction with outcome of treatment,^4,8^ risk of pain,^4,8,23^ risk of prolonged rehabilitation period,^23^ survival time at expense of quality of life,^23^ effect of mastectomy on intimacy,^28^ concern for disfigurement,^10,29,30^ remove breast for peace of mind,^20^ avoid need for future disease screening,^20^ fertility risk,^31^ negative impact on sexual life,^9,10^ fear of losing partners,^9^ shock of diagnosis^16,18,32^ |
| Adherence to Treatment and Compliance | Treatment Refusal  - | Decide against undergoing treatment,^31^ long travel distances leading to questionable compliance and frequent treatment abandonment^30^ |
| Physician Professional Background^33^ | Experience,^3,33^ Position within Department | Level of education of physician,^13^ years working as a specialist^13^ |
| Professional Interaction/  Communication  *Amongst all stakeholders* | Physician time constraints,^8,21,25,34^ patient/caregiver participation in decision making,^4,6,8,11,35,36^ patient/caregiver trust in healthcare system^7,16,17,19,25,37^ | Patient/family time spent with physician,^4^ patient-physician communication,^37-39^ obtaining second opinion,^9,20,25^ patient-doctor interactions,^4,8^ access to ethics consultant,^3^ decision made by patient’s husband (related to breast cancer),^4,5,8^ patriarchal society,^40^ need to acquiesce to authority (fear of raising questions may lead to receiving poorer care),^31^ deliberate miscommunications of treatment intent (goal to prevent woman from refusing treatment),^31^ patients disempowered,^31^ degree of engagement/ability of parents to participate in decision making^19^ |
| **Decision Specific Factors** | | |
| Patient Characteristics^41^ | Age,^11,12,15,26,33,42,43^ Gender, Comorbidities,^33,38,42-44^ Performance Status^12,26,43,45-47^ | - |
| Treatment Toxicities^7,18,26,27,30,39,47-51^ | - | Adverse effects of treatment,^24,25,39^ risks of therapy or surgery,^25^ unwanted side effects,^3^ effect of surgery on functional status,^2^ patient’s tolerance of treatment,^52^ concerns about impact on fertility^31^ |
| Disease Characteristics^12^ | Tumor Stage^4,20,32,38,41,42,45,53-55^  Delayed Presentation  Morphological/Histological Features^12^ |  |
| Diagnostics | Biomarker/Laboratory Values  Access to Diagnostics^53,56^ | - |
| Treatment Intent/Anticipated Outcome of Treatment | Time Pressure (to treat the disease)^41^ | Prognosis,^19,26,38,45^ likelihood of treatment benefit,^19,26,27,48^ prevention of disease recurrence,^17,20,51^ expected outcome of treatment,^34^ potential treatment benefit,^57^ therapeutic intent,^57^ survival potential/curability,^2,24,49,52,55,58^ disease control,^24,49^ efficacy of treatment,^3,11^ potential risks of delaying treatment,^34,52^, response to treatment^50^ |
| COVID-19 and other infectious diseases | - | COVID-19,^12,38,41,45,52,55,57,59-61^ Tuberculosis^62^ |
| Treatment and Supportive Care^38^ | Medication Quality/Supply, Availability of Various Therapeutic Options,^48,53^ Access to Treatment and Supportive Care,^8,25,30,31,33-35,37,45,50,53,55,56^ Access to Trained Workforce,^30,31,56^ Treatment Recommendation^3,4,9,10,19,25,31,36,48,51,63^ | Uncertainty about best treatment option,^25^ waiting period for radiation therapy,^8^ resource limitations,^30^ inadequate infrastructure,^30^ insufficient facilities/equipment,^21^ access to staff/human resources to guarantee good outcome,^34^ lack of clinical ethics counseling^3^ |
| **Contextual Factors** | | |
| Practice Setting/Organization | - | Referral pathway,^64^ organization of healthcare system,^64^ private hospital,^53^ public hospital,^53^ tertiary center,^11^ policies related to ethics,^3^ academic setting,^13^ hospital protocols and bureaucracies,^37^ location of practice,^11^ workplace^3^ |
| Government Policies/Political Climate | Political Climate,^56^ Physical Environment | Destroyed facilities and blocked roads due to conflict,^56^ war, visa regulations,^56^ corruption,^34^ political factors,^34^ national and institutional policies surrounding, legal concerns regarding withholding or withdrawing futile treatment,^3^ vague national and institutional policies^3^ |
| Socioeconomic Status^4,11^ | Health Literacy,^8,31^ Financial Situation,^6,7,16,18,31,36,39,40,48,49,54,65-68^ Access to Healthcare,^19,34,56^ Ability to Seek Care Abroad^7^ | Economic burden of treatment,^34,48,58^ poverty,^48^ ability to transport patient body home after death if death occurred in different city,^48^ ability to travel within country to access healthcare/treatment,^34,48^ risk associated with travel,^48^ patient/caregiver perception of cancer and cancer treatment,^18^ awareness and acceptability of treatment,^1^ misperceptions about surgical risks in management of disease,^1^ poor patient will turn to integrative or traditional medicine,^7^ husband paying for treatment, education level,^11,54,67^ knowledge about breast cancer,^28^ misperceptions,^1^ employment^4,5,67^ |
| Culture/Religion^3,33,37^ | Preference for Traditional Medicine^7,30,48^ | Stigma,^1,18,30,69^ myths,^1^ cultural beliefs and values,^1,11,18,23,69,70^ superstitions,^1,37^ health beliefs,^69^ need to consult cultural decision maker who may not have insight into patient's condition,^69^ local traditional belief system,^37^ traditional healer directive,^37^ destiny,^37^ spiritual origins of cancer,^31^ language barriers and limitations in local language to describe cancer/cancer therapies,^31^ gender bias (neglect of female children),^30^ treatment culture in the community,^7^ faith,^28^ spirituality^18^ |
| Access to Resources/  Information^16^  *To support decision-making* | - | Access to treatment protocols,^53^ use of clinical practice guideline,^21^ feasibility of using clinical practice guideline,^21^ access to guidelines adaptable for local content,^21^ access to tumor board,^21,35,53,71-73^ access to multidisciplinary team,^48^ most physicians seem to make complicated decisions about patient referral only after group or departmental discussions with their physician colleagues,^48^ inability to discuss research with knowledgeable colleagues,^21^ sources of information patient relies on to make treatment decision such as medical team, internet, books, television, friends,^74^ the parents of other children who were staying in the hospital^16^ |
| Influence of Family/Community^3,9,10,19,23,25,26,28,32,37,40,63-65,74^  *Includes caregivers* | Access to Family Support System,^18,48^ Desire to Help/Support Other Patients^17^ | Roles and responsibilities in the family,^7^ cancer treatment prevents patient from participating in their role and function in the family,^7^ testimonies of other cancer patients or survivors,^9,17,74,75^ influence of husband,^4,5^ influence of male family members,^40,67^ familial obligation to treat family member curatively,^23^ assessment by the relatives that they patient was too weak/old to tolerate any major procedure,^29^ opinions of the public,^37^ external sources influence patient decisions: cancer survivor, social media, involvement of health workers^7^ |
| Reimbursement Policies^3,4,51^/  Cost of Treatment^2,3,7,11,18,23,25,26,29-32,34,40,47,49-51,53,58,76,77^ | - | Out-of-pocket costs for patients and caregivers,^24,30^ access to health insurance,^30,33,34,54,66^ costs associated with treatment^56^ |
| Resource Allocation^38,41,45,52,53,55,78,79^ | - | - |

Supplemental Table 4. Summary of articles by theme and factor.

| **Theme** | **Factor** | **No. of articles (n)** | **No. of articles (%)** |
| --- | --- | --- | --- |
| **Decision Maker** | Personal Belief System/Characteristics | 24 | 30 |
|  | Quality of Life (Patient) | 21 | 27 |
|  | Risk of Non-Compliance/Treatment Refusal | 5 | 16 |
|  | Physician Professional Background | 5 | 16 |
|  | Professional Interaction/Communication | 20 | 25 |
| **Decision Specific** | Patient-Related Features | 12 | 15 |
|  | Treatment Toxicities | 25 | 32 |
|  | Features of Cancer | 12 | 15 |
|  | Diagnostics | 4 | 5 |
|  | Treatment Intent/Anticipated Outcome | 27 | 34 |
|  | COVID-19 and other Infectious Diseases | 9 | 11 |
|  | Treatment and Supportive Care | 31 | 40 |
| **Contextual** | Practice Setting/Organization | 6 | 8 |
|  | Government Policies/  Political Climate | 3 | 4 |
|  | Socioeconomic Status | 32 | 41 |
|  | Culture/Religion | 13 | 16 |
|  | Access to Resources/Information | 10 | 13 |
|  | Influence of Family/Community | 25 | 32 |
|  | Reimbursement Policies/Cost of Treatment | 31 | 40 |
|  | Resource Allocation | 8 | 10 |
| **Articles reporting factors:** | In only 1 domain | 27 | 34 |
|  | In 2 domains | 23 | 29 |
|  | Across three domains | 29 | 37 |

References

1. Pankaj S, Nazneen S, Kumari A, et al: Myths and Taboos- A Major Hindrance to Cancer Controls. “Inherited Knowledge” A Blessing or Curse. Surgery After 21 Cycles of Chemotherapy “A Surgeon’s Ordeal". Indian Journal of Gynecologic Oncology 16, 2018

2. Zhang L, Jiang M, Zhou Y, et al: Survey on breast cancer patients in China toward breast-conserving surgery. Psychooncology 21:488-95, 2012

3. Demir Kureci H, Tanriverdi O, Ozcan M: Attitudes towards and experiences of ethical dilemmas in treatment decision-making process among medical oncologists. J Eval Clin Pract 26:209-215, 2020

4. Agrawal S, Goel AK, Lal P: Participation in decision making regarding type of surgery and treatment-related satisfaction in North Indian women with early breast cancer. J Cancer Res Ther 8:222-5, 2012

5. Lin YP, Chen SZ, Yin WJ, et al: Factors that influencing patients' decision making for breast conserving surgery. Fudan University Journal of Medical Sciences 25:641-645, 2008

6. Behan JM, Arora RS, Carnevale FA, et al: An Ethnographic Study of the Moral Experiences of Children with Cancer in New Delhi, India. Global Qualitative Nursing Research 8:1-14, 2021

7. Deliana M, Suza DE, Tarigan R: Advanced Stage Cancer Patients Experience in Seeking Treatment in Medan, Indonesia. Open Access Maced J Med Sci 7:2194-2203, 2019

8. Mishra A, Agarwal R, Tewari S, et al: Factors in Decision Making of Breast Conservation in Early Breast Cancer: a Study in Northern India. European Journal of Cancer 48, 2012

9. Yuksel E, Guven HE, Dogan L: Patients' Perspective: What has Changed in Deciding about Breast-Conserving Surgery for Early-Stage Breast Cancer in Turkey? Oncol Res Treat 41:744-749, 2018

10. Teh YC, Shaari NE, Taib NA, et al: Determinants of Choice of Surgery in Asian Patients with Early Breast Cancer in a Middle Income Country. Asian Pac J Cancer Prev 15:3163-7, 2014

11. Doval DC, Kumar P, Talwar V, et al: Shared Decision-Making and Medicolegal Aspects: Delivering High-Quality Cancer Care in India. Indian J Palliat Care 26:405-410, 2020

12. Batistella GNR, Santos AJ, Paiva Neto MA, et al: Approaching glioblastoma during COVID-19 pandemic: current recommendations and considerations in Brazil. Arq Neuropsiquiatr 79:167-172, 2021

13. Wu X, Jiang YN, Zhang YL, et al: Impact of Physicians' Personalities and Behavioral Traits on Treatment-Related Decision-making for Elderly Acute Myeloid Leukemia. J Gen Intern Med 36:3023-3030, 2021

14. Wu X, Jiang Y-N, Zhang Y-L, et al: Impact of Hematologists' Personality and Behavioral Traits on Medical Decision-Making for Elderly Acute Myeloid Leukemia: A National Study in China. Blood 136:19-19, 2020

15. Gumus M, Ustaalioglu BO, Garip M, et al: Factors that Affect Patients' Decision-Making about Mastectomy or Breast Conserving Surgery, and the Psychological Effect of this Choice on Breast Cancer Patients. Breast Care (Basel) 5:164-168, 2010

16. Kilicarslan-Toruner E, Akgun-Citak E: Information-seeking behaviours and decision-making process of parents of children with cancer. Eur J Oncol Nurs 17:176-83, 2013

17. Shariff Z, Mansor, AZ, Muhamad, M: Decision Making in Breast Cancer Treatment - A Qualitative Inquiry. Pertanika Journal of Social Sciences and Humanities 16:269-278, 2008

18. Ogunkorode A, Holtslander L, Ferguson L, et al: Factors influencing the health-seeking behaviors of women with advanced stages of breast cancer in Southwestern Nigeria: An interpretive description study. International Journal of Africa Nursing Sciences 14, 2021

19. De Guzman BG, Cabaya NF, Ting FIL, et al: Factors influencing treatment decisions among breast cancer patients in the Philippine general hospital cancer institute: Medical oncology outpatient clinic. Annals of Oncology 30, 2019

20. Obeidat RF, Masri MA, Marzouq M: Factors Affecting Jordanian Women's Surgical Treatment Decisions for Early-Stage Breast Cancer. Asia Pac J Oncol Nurs 8:711-719, 2021

21. Bhandari D, Ozaki A, Ghimire B, et al: Oncology clinical practice guidelines usage among physicians in Nepal. J Eval Clin Pract 28:142-150, 2022

22. El-Hadidy MA, Elnahas W, Hegazy MA, et al: Psychiatric morbidity among Egyptian breast cancer patients and their partners and its impact on surgical decision-making. Breast Cancer (Dove Med Press) 4:25-32, 2012

23. Gong N, Du Q, Lou H, et al: Treatment decision-making for older adults with cancer: A qualitative study. Nursing Ethics 28:242-252, 2021

24. Zhou N, Liu F, Hu M, et al: Patient Preference Study on Treatments of Non Small Cell Lung Cancer in Western China. Value in Health 21, 2018

25. Lee PL, Cheong AT, Ng CJ, et al: Supporting patients in making treatment decisions for early prostate cancer - A qualitative study of healthcare professionals' views on barriers and challenges in an Asian country. Journal of Men’s Health 12:18-24, 2016

26. Gielen J, Bhatnagar S, Mishra S, et al: Can curative or life-sustaining treatment be withheld or withdrawn? The opinions and views of Indian palliative-care nurses and physicians. Med Health Care Philos 14:5-18, 2011

27. Salek M, Force LM, Hlatwayo L, et al: An Approach to Understanding Clinician Treatment Decision-Making at Diagnosis in Pediatric Cancer: Exploring Challenges Faced by Clinicians in Zimbabwe. Pediatr Blood Cancer 68:S346, 2021

28. Aziato L, Clegg-Lamptey JN: Breast cancer diagnosis and factors influencing treatment decisions in Ghana. Health Care Women Int 36:543-57, 2015

29. Ramakrishnan V, Kirushnakumar KS, Rathinam K, et al: Factors affecting treatment options in patients with advanced cancer of oral cavity - a single institution prospective study from Southern India. Asia-Pac J Clin Oncol 8:294-295, 2012

30. Mailankody S, Kumar VS, Khan SA, et al: Resource-appropriate selection of osteosarcoma treatment protocols in low- and middle-income countries. Pediatr Blood Cancer 69:e29540, 2022

31. Agyemang LS, Foster C, McLean C, et al: The cultural and structural influences that 'hide' information from women diagnosed with breast cancer in Ghana: an ethnography. BMC Womens Health 21:364, 2021

32. Tang L: Barriers to effective decision making in cancer patient. Asia-Pac J Clin Oncol 8:297, 2012

33. Hurdle V, Ouellet JF, Dixon E, et al: Does regional variation impact decision-making in the management and palliation of pancreatic head adenocarcinoma? Results from an international survey. Can J Surg 57:E69-74, 2014

34. Kowalski LP, Sanabria A: Priority setting in head and neck oncology in low-resource environments. Curr Opin Otolaryngol Head Neck Surg 27:198-202, 2019

35. Rosabal-Obando M, Osorio DS, Lassaletta A, et al: Follow-up evaluation of a web-based pediatric brain tumor board in Latin America. Pediatr Blood Cancer 68:e29073, 2021

36. Ahmadnia S, Ghalibaf AK, Kamkar S, et al: Survivor and parent engagement in childhood cancer treatment in Iran. Ecancermedicalscience 15:1220, 2021

37. Salisu WJ, Mirlashari J, Seylani K, et al: Fatalism, distrust, and breast cancer treatment refusal in Ghana. Can Oncol Nurs J 32:198-205, 2022

38. Al-Tabba A, Al-Hussaini M, Mansour R, et al: Ethical Considerations for Treating Cancer Patients During the SARS-CoV-2 Virus Crisis: To Treat or Not to Treat? A Literature Review and Perspective From a Cancer Center in Low-Middle Income Country. Front Med (Lausanne) 7:561168, 2020

39. Wang T, Molassiotis A, Chung BPM, et al: A qualitative exploration of the unmet information needs of Chinese advanced cancer patients and their informal caregivers. BMC Palliat Care 20:83, 2021

40. Alexander A, Kaluve R, Prabhu JS, et al: The Impact of Breast Cancer on the Patient and the Family in Indian Perspective. Indian J Palliat Care 25:66-72, 2019

41. Jain A, Singh C, Dhawan R, et al: How to Use a Prioritised Approach for Treating Hematological Disorders During the COVID-19 Pandemic in India? Indian J Hematol Blood Transfus 36:605-615, 2020

42. Menon MP, Coghill A, Mutyaba I, et al: Treatment Recommendations for Patients with NHL at the Uganda Cancer Institute. Blood 122:2960-2960, 2013

43. Pawar D, Ahmed R, Chaudhari S: A survey of on platinum ineligible in head and neck cancer patients in India. Annals of Oncology 28, 2017

44. Baijal G, Gupta T, Hotwani C, et al: Impact of comorbidity on therapeutic decision-making in head and neck cancer: audit from a comprehensive cancer center in India. Head Neck 34:1251-4, 2012

45. Bhatla N, Singhal S: The COVID-19 Pandemic and Implications for Gynaecologic Cancer Care. Indian J Gynecol Oncol 18:48, 2020

46. Peruzzo N, Coelho JE, Gossling G, et al: Treatment delay and outcomes in stage IV lung cancer: The reality of a public hospital in a developing country. Journal of Clinical Oncology 37, 2019

47. Ngorsuraches S, Thongkeaw K: Patients' preferences and willingness-to-pay for postmenopausal hormone receptor-positive, HER2-negative advanced breast cancer treatments after failure of standard treatments. Springerplus 4:674, 2015

48. Harris JJ, Shao J, Sugarman J: Disclosure of cancer diagnosis and prognosis in Northern Tanzania. Social Science & Medicine 56:905-913, 2003

49. Zhou N, Liu F, Jing W, et al: Study on Physicians' Preference for Treatment of NSCLC in Western China. Value in Health 22:S111-S112, 2019

50. Malhotra P, Yanamandra U, Kumar S, et al: Common Reasons for Change of Chemoregimens in Multiple Myeloma: Real World, Comparison of Two Tertiary Care Centers. Clinical Lymphoma Myeloma and Leukemia 19, 2019

51. Sun H-C, Zhu X-D, Xu L, et al: Factors influencing adjuvant treatment decision making among Chinese patients with hepatocellular carcinoma (HCC): Results of a patient survey. Journal of Clinical Oncology 39:346-346, 2021

52. Ramesh A, Ssoundarajan R: Cancer care during the COVID-19 pandemic in Southern India. Clinical Cancer Research 26:PO-008-PO-008, 2020

53. Pereira da Veiga CR, Pereira da Veiga C, Drummond-Lage AP, et al: Journey of the Patient With Melanoma: Understanding Resource Use and Bridging the Gap Between Dermatologist, Surgeon, and Oncologist in Different Health Care Systems. J Glob Oncol 5:1-8, 2019

54. Liu JJ, Zhang S, Hao X, et al: Breast-conserving therapy versus modified radical mastectomy: socioeconomic status determines who receives what--results from case-control study in Tianjin, China. Cancer Epidemiol 36:89-93, 2012

55. Del Pilar Estevez-Diz M, Bonadio RC, Miranda VC, et al: Management of cervical cancer patients during the COVID-19 pandemic: a challenge for developing countries. Ecancermedicalscience 14:1060, 2020

56. Skelton M, Alameddine R, Saifi O, et al: High-Cost Cancer Treatment Across Borders in Conflict Zones: Experience of Iraqi Patients in Lebanon. JCO Glob Oncol 6:59-66, 2020

57. Mendoza MJL, Tan HNC, Hernandez ARB, et al: Medical oncology care amidst the COVID-19 pandemic at the National University Hospital in the Philippines. Ecancermedicalscience 14:1066, 2020

58. Hong D, Zhou C, He H, et al: A 10-Year Follow-up Survey of Treatment Abandonment of Children With Acute Myeloid Leukemia in Suzhou, China. J Pediatr Hematol Oncol 38:437-442, 2016

59. Siavashpour Z, Taghizadeh-Hesary F, Rakhsha A: Recommendations on Management of Locally Advanced Rectal Cancer During the COVID-19 Pandemic: an Iranian Consensus. J Gastrointest Cancer 51:800-804, 2020

60. Vanderpuye V, Elhassan MMA, Simonds H: Preparedness for COVID-19 in the oncology community in Africa. The Lancet Oncology 21:621-622, 2020

61. Pritchard-Jones K, Abib S, Esiashvili N, et al: The threat of the COVID-19 pandemic on reversing global life-saving gains in the survival of childhood cancer: a call for collaborative action from SIOP, IPSO, PROS, WCC, CCI, St Jude Global, UICC and WHPCA. ecancer 15:1187, 2021

62. Nair CK, Avaronnan M, Shenoy PK, et al: Impact of active tuberculosis on treatment decisions in cancer. Curr Probl Cancer 45:100643, 2021

63. Muhamad M, Afshari M, Kazilan F: Family Support in Cancer Survivorship. Asian Pacific J Cancer Prev 12:1389-1397, 2011

64. Agom DA, Allen S, Neill S, et al: Social and Health System Complexities Impacting on Decision-Making for Utilization of Oncology and Palliative Care in an African Context: A Qualitative Study. J Palliat Care 35:185-191, 2020

65. Datta SS, Tripathi L, Varghese R, et al: Pivotal role of families in doctor-patient communication in oncology: a qualitative study of patients, their relatives and cancer clinicians. Eur J Cancer Care (Engl) 26, 2017

66. Li X, Dong M, Wen J-y, et al: Cancer patients’ awareness and role in family-based medical decision-making mode in Confucian area. Journal of Clinical Oncology 32:227-227, 2014

67. Alexander A, Kaluve R, Prabhu JS, et al: Treatment decision making, and strategies for coping with financial stress in Indian women diagnosed with breast cancer and their families. Cancer Research 78:P4-10-12-P4-10-12, 2018

68. Soltani L, Khoshnood Z: Social support needs in patients with cancer. Middle East Journal of Cancer 12:429-438, 2021

69. Brown O, Goliath V, van Rooyen DRM, et al: Cultural factors that influence the treatment of osteosarcoma in Zulu patients: Healthcare professionals' perspectives and strategies. Health SA 23:1095, 2018

70. Brucker ME: Cultural considerations in palliative care-oncology healthcare decision making- An observational study of familial influence in India and other cultures. Cancer Nursing 38:S52-S53, 2015

71. Abbasi AN, Qureshi BM, Karim MU: Impact of Multidisciplinary Team Meetings and Decision-Making on Cancer Management in Lower and Middle Income Countries. Chest 159:887-888, 2021

72. Amaro CP, Gomes L, Almeida D, et al: Impact of multidisciplinary discussion on therapeutic decision in cancer patients: a prospective observational study. Journal of Clinical Oncology 35:e18243-e18243, 2017

73. Kouya F, Picton S, Squire R, et al: Establishing a Virtual Multidisciplinary Team Meeting between Cameroon, Central Africa, and Leeds, UK, Childhood Cancer Services. Pediatr Blood Cancer:S68-S69, 2021

74. Abdullah A, Abdullah KL, Yip CH, et al: The decision-making journey of Malaysian women with early breast cancer: a qualitative study. Asian Pac J Cancer Prev 14:7143-7, 2013

75. Olasehinde O, Arije O, Wuraola FO, et al: Life Without a Breast: Exploring the Experiences of Young Nigerian Women After Mastectomy for Breast Cancer. J Glob Oncol 5:1-6, 2019

76. Daroudi R, Mirzania M, Zendehdel K: Attitude of Iranian Medical Oncologists Toward Economic Aspects, and Policy-making in Relation to New Cancer Drugs. Int J Health Policy Manag 5:99-105, 2015

77. Bhattacharya M, Hamilton EP, Zafar Y: Oncologists’ perceptions of cost and cancer care in India: A comparison of private practice (PPOs) and non-private practice oncologists (NPPOs). Journal of Clinical Oncology 31:e17562-e17562, 2013

78. Shelal Z, Alawad AS, Sun C, et al: Practicing oncology amidst war and religious strife-how do physician beliefs affect care and work-related stress? Support Care Cancer 20 Suppl 1:S196, 2012

79. Yousef MH, Alhalaseh YN, Mansour R, et al: The Fair Allocation of Scarce Medical Resources: A Comparative Study From Jordan. Front Med (Lausanne) 7:603406, 2020
